# Magnetically driven active topography for long-term biofilm control

**DOI:** 10.1038/s41467-020-16055-5

**Published:** 2020-05-05

**Authors:** Huan Gu, Sang Won Lee, Joseph Carnicelli, Teng Zhang, Dacheng Ren

**Affiliations:** 1 Department of Biomedical and Chemical Engineering, Syracuse University, 329 Link Hall, Syracuse, New York 13244 USA; 2 Syracuse Biomaterials Institute, Syracuse University, 318 Bowne Hall, Syracuse, New York 13244 USA; 3 Department of Mechanical and Aerospace Engineering, Syracuse University, 214 Link Hall, Syracuse, New York 13244 USA; 4 Department of Civil and Environmental Engineering, Syracuse University, 151 Link Hall, Syracuse, New York 13244 USA; 5 Department of Biology, Syracuse University, 114 Life Sciences Complex, Syracuse, New York 13244 USA

**Keywords:** Biomedical materials, Implants, Biomaterials - cells

## Abstract

Microbial biofilm formation on indwelling medical devices causes persistent infections that cannot be cured with conventional antibiotics. To address this unmet challenge, we engineer tunable active surface topographies with micron-sized pillars that can beat at a programmable frequency and force level in an electromagnetic field. Compared to the flat and static controls, active topographies with the optimized design prevent biofilm formation and remove established biofilms of uropathogenic *Escherichia coli* (UPEC), *Pseudomonas aeruginosa*, and *Staphylococcus aureus*, with up to 3.7 logs of biomass reduction. In addition, the detached biofilm cells are found sensitized to bactericidal antibiotics to the level comparable to exponential-phase planktonic cells. Based on these findings, a prototype catheter is engineered and found to remain clean for at least 30 days under the flow of artificial urine medium, while the control catheters are blocked by UPEC biofilms within 5 days.

## Abstract (web summary)

Biofilm formation is a major problem in indwelling medical devices. Here, the authors report on the development of a magnetically responsive micro pillar surface for the controlled prevention and removal of biofilms which also increased sensitivity to antibiotics.

## Introduction

According to the Centers for Disease Control and Prevention (CDC), there are 1.7 million cases of hospital-acquired (nosocomial) infections with 99,000 associated deaths annually in the U.S. alone. In addition to the medical challenges, the treatment of nosocomial infections costs 20 billion dollars in the U.S. each year^1^. Urinary tract infections (UTIs) are among the most common types of healthcare-associated infections, and 75% of UTIs involve a urinary catheter^2^. The leading cause of catheter-associated UTIs (CAUTIs) is prolonged use, which increases exposure to microbial pathogens. Both bacteria and fungi can attach to implanted medical devices using flagella, pili, and other factors such as adhesins^3^. The attachment of microbes leads to the subsequent formation of a biofilm, which is a surface-attached multicellular structure comprised of an extracellular matrix secreted by the attached cells^4–6^. Biofilm-associated infections are difficult to treat because of the extremely high tolerance of biofilm cells to antimicrobials (up to 1,000 times higher compared to their planktonic counterparts)^4,5,7^. The close contact between biofilm cells also provides an ideal environment for bacterial horizontal gene transfer through conjugation, a process that leads to the emergence of multidrug-resistant bacteria, including superbugs^8^. Thus, novel devices and materials for effective control and prevention of biofilm-associated infections are urgently needed.

Biofilm formation is a dynamic process affected by many properties of the substratum material, such as surface chemistry^9,10^, stiffness^11,12^, hydrophobicity^13,14^, roughness^15,16^, topography^17–20^, and charge^21,22^. Previous research revealed that plants and animals had developed antifouling surface topographies, which can be mimicked to reduce biofouling^23–26^. Because these antifouling topographies do not require antimicrobials, this strategy will not promote the development of antimicrobial resistance associated with chemical approaches, e.g., antimicrobial coating of medical devices^27^. Recently, we reported a set of principles for the rational design of micron-scale antifouling topographies^28^. Besides micron-scale topographies, nano-scale topographies such as those on the wings of cicadas and dragonflies also have antimicrobial activities and can be mimicked for fouling control^25^. However, static topographies have limitations in long-term fouling control, because the small number of cells that attach can grow and gradually overcome the unfavorable topographies^29^.

Motivated by these challenges, we aim to develop strategies with sustained antifouling activities. We hypothesize that by controlling the synchronized movement of micron-scale topographic features, enough force can be generated to repel bacteria from attaching to a surface and remove established biofilms on demand. Antifouling systems based on active topographies exist in nature; e.g., human lung epithelial cells repel microbes with beating cilia and thus prevent infection^30^. Some studies have been conducted to mimic cilia for generating local motion and mechanoreception^31–35^. However, these designs are not applicable to fouling control in vivo due to limitations of the actuation mechanisms, which include the use of large magnets^31,32^, narrow working temperature ranges^33,34^, or supply of specific chemicals (such as ATP)^35^. In addition, previous studies used the rotational movement of a magnetic field (e.g., with a stirring plate) to drive the pillars, which is different from the escalating beats of cilia in vivo^30,36^. More importantly, human motile cilia propel a superficial mucus layer that entraps foreign particles and pathogens to remove them out of the airway by beating in an underlying periciliary layer^30^. Both the mucus and periciliary layers are essential for the antifouling effects of cilia^30^, but missing in the application environments of most medical devices, e.g., urinary catheters.

To address these unmet challenges, we report a platform technology with active surface topographies based on micron-scale pillars driven by a programmable electromagnetic field. Instead of using bulky equipment, this design uses a metal coil to generate the magnetic field, which eases the integration into medical devices. Our data indicate that effective long-term fouling control (more than 30 days in a prototype catheter) can be achieved without mucin. The beating force and frequency of these pillars are tunable, and the fabrication process is scalable for different polymers.

## Results

### Engineering magnetically responsive micron-sized pillars

**Fig. 1:**
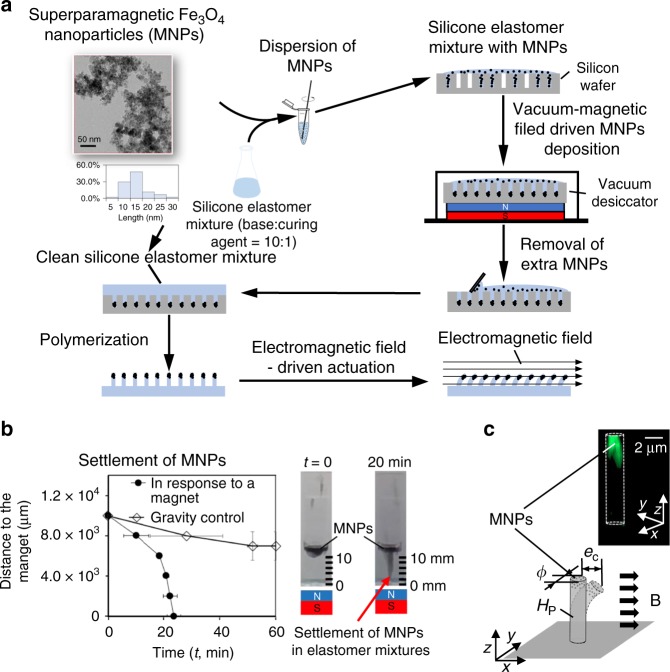
Engineering magnetically responsive PDMS pillars. **a** Schematic of the steps for fabricating active surface topographies. **b** Settlement of MNPs through silicone elastomer mixture (base: curing agent = 10:1) driven by a 1 T magnet. The images show the original MNPs in a PDMS mixture and the settling of MNPs with a magnet. Each condition was tested with three replicates (error bar = standard deviation; *n* = 3). Source data of Fig. 1a, b are provided in the Source Data file. **c** Schematic of pillar bending in response to an external electromagnetic field. The location of MNPs in the pillar tips is indicated in the schematic, which was verified by imaging pillars loaded with fluorescently labeled MNPs (green signals shown in Fig. 1c). The profile of the pillar is highlighted with white dotted lines.

In this study, we engineered a programmable antifouling system using active surface topographies that can be actuated by external stimuli with tunable beating frequency and force. This was achieved by loading superparamagnetic Fe_3_O_4_ nanoparticles (MNPs) at the tip of each polymer pillar using a magnet and vacuum during lithography (Fig. 1a, b). The size of MNPs was determined to be 12.5 ± 4.7 nm (mean ± standard deviation) using transmission electron microscopy (TEM, Fig. 1a); and the loading of MNPs at the tips of pillars was confirmed using scanning electron microscopy and energy dispersive X-ray spectroscopy (SEM-EDS) (Supplementary Fig. 1), and microscopy with fluorescently tagged MNPs (Fig. 1c). When a magnetic field is applied perpendicularly to a pillar, the MNPs will move, generating a force that causes the pillar to bend (Fig. 1c). With quick removal of the magnetic field, the pillar returns to its original position due to intrinsic material elasticity (Fig. 1c). Thus, oscillation (on/off) of this external magnetic field drives repeated beating of the pillars. Because the fabrication is based on lithography, such active topographies can be easily created with a wide range of materials. In this first study, we chose poly(dimethylsiloxane) (PDMS) because it is a biocompatible polymer widely used in medical devices, including urinary catheters^37^.

With tunable beating force and frequency, active topography brings an opportunity to achieve both biofilm prevention (by repelling bacteria) and removal of mature biofilms (with stronger forces to disrupt the biofilm structure). We started with cylindrical topographic features with a diameter (*ϕ*) of 2 µm, inter-pillar distance (*D*) of 2 µm, height (*H*_P_) of 10 µm, and Young’s modulus (*E*_P_) of 2.1 MPa. The height of 10 µm was chosen because it is the length of human cilia^36,38^ and can prevent bacterial flagella (6–10 µm long^39^) from reaching the grooves between pillars for adhesion^28^. Meanwhile, the size and distribution of pillars were selected to ensure enough flexibility for the pillars to bend. The degree of pillar deformation (*e*_c_) was adjusted by tuning the strength of the electromagnetic field (**B**; see Supplementary Methods 1.1 for details). Biofilm formation on a surface involves four major steps that are initial attachment, microcolony formation, maturation, and dispersion^6^. Early attached cells are in a monolayer and rather sensitive to environmental stresses, while mature biofilms have three-dimensional (3D) structures and are more tolerant to such stresses^6^. To understand if active topographies are effective in controlling biofilms at different stages of development, we tested three modes of actuation, including continuous actuation for biofilm prevention (1 mT), on-demand actuation for biofilm removal (5 mT for stronger forces), and a sequential combination of the two treatments. The frequency of beating was kept at 10 Hz for all tests, which is comparable to the beating frequency of human motile cilia in the respiratory system (15 Hz)^36,38^.

### Antifouling effects against UPEC biofilms

**Fig. 2:**
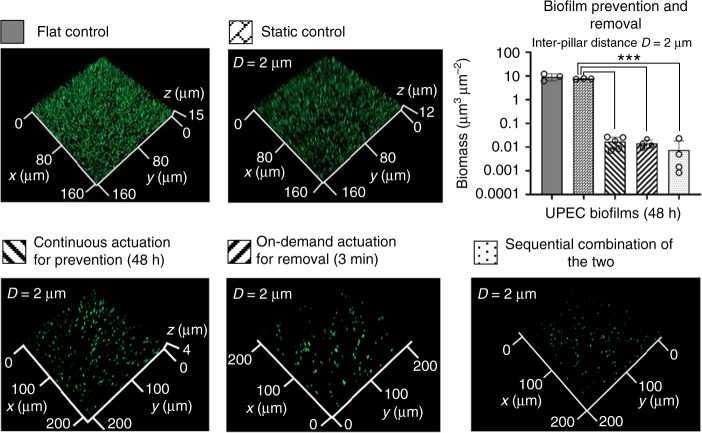
Active pillars exhibited profound antifouling effects against UPEC biofilms. Representative fluorescence images of biofilms on flat controls, static controls, and PDMS surfaces with active topographies. The pillars were 10 µm tall with a diameter of 2 µm and inter-pillar distance of 2 µm. Active surface topographies were operated in three modes for biofilm prevention and removal, including continuous beating for biofilm prevention, on-demand removal of mature biofilms (only actuated for 3 min after 48 h of biofilm growth), and a sequential combination of these two treatments. Biofilm cells were labeled with STYO^®^9. The biomass was quantified using COMSTAT^59^. The samples in the bar graph are indicated with the pattern labels above the corresponding fluorescence images. Each condition was tested with at least three biological replicates (error bar = standard deviation; *n* = 3–5; ***p < 0.0001, one-way ANOVA adjusted by Tukey test), and five random images were taken from each sample. Source data of Fig. 2 are provided in the Source data file.

The antifouling effects of engineered active topographies were first evaluated against the biofilm formation of uropathogenic *Escherichia coli* (UPEC), the primary causative agent of CAUTI^40^. As expected, the beating of pillars demonstrated potent effects in preventing biofilm formation and disrupting mature biofilms of a UPEC strain ATCC53505 (Fig. 2). Specifically, continuous actuation at 1 mT kept the PDMS surface clean throughout the test (48 h) with only 0.02 ± 0.01 µm^3^ µm^−2^ cells attached at the end of the experiment (Fig. 2). This biomass is 2.6 logs (99.8%) less than that on static control surfaces (with the same pillars but without actuation) (7.6 ± 0.3 vs. 0.02 ± 0.01 µm^3^ µm^−2^; *p* < 0.0001, one-way ANOVA adjusted by Tukey test) and 2.7 logs (99.8%) less than that on flat PDMS surfaces (flat controls) (9.2 ± 3.0 vs. 0.02 ± 0.01 µm^3^ µm^−2^; *p* = 0.0003). To obtain more insights into biofilm inhibition by active topography, we also imaged the surfaces at 3 and 6 h after inoculation. The images in Supplementary Fig. 2 showed that biomass increased over time for both the flat and static controls, while active topography reduced early biofilm formation and kept the biomass low throughout the 48-h test. Strong effects were also observed for the removal of mature biofilms (cultured for 48 h without actuation of the pillars first) by activating pillars on-demand at 5 mT for 3 min. This treatment caused 2.7-log (99.7%) reduction of biomass compared to the static control (7.6 ± 0.3 vs. 0.01 ± 0.006 µm^3^ µm^−2^; *p* < 0.0001). When the pillars were actuated during biofilm formation followed by stronger beating for biofilm removal, the sequential treatment reduced biofilm biomass by 3.0 logs (7.6 ± 0.3 vs. 0.008 ± 0.01 µm^3^ µm^−2^; *p* < 0.0001) compared to the static control and 3.1 logs compared to the flat control (9.2 ± 3.0 vs. 0.008 ± 0.01 µm^3^ µm^−2^; *p* = 0.0013) (Fig. 2). This doubled the effect on biofilm dispersion achieved by on-demand actuation alone (e.g., 3.0 vs. 2.7 logs of reduction compared to the static control).

### Optimization of pillar design

To understand if the antifouling effects are species-specific, we repeated the experiment with biofilm formation of *Pseudomonas aeruginosa* and *Staphylococcus aureus*. These two species were chosen because of their significance in medical device-associated infections and different mechanisms of biofilm formation^6,7^. Like UPEC results, active topography also inhibited biofilm formation of *P. aeruginosa* PAO1 and *S. aureus* ALC2085 (*D* = 2 µm; Supplementary Figs. 3, 4). However, during the first 48 h of biofilm formation, continuous actuation of active pillars gradually lost its effects in preventing *S. aureus* biofilm formation as the biomass reduction (compared to the flat control) decreased from 2.4 logs at 3 h to 0.8 log at 48 h after inoculation (Supplementary Fig. 4). We also observed more biofilm cells trapped between pillars with narrow spacing (2 µm) compared to UPEC results (Supplementary Tables 1–3; Supplementary Figs. 5, 6). We speculate that there is a critical inter-pillar distance for an optimal, broad-spectrum antifouling effect.

**Fig. 3:**
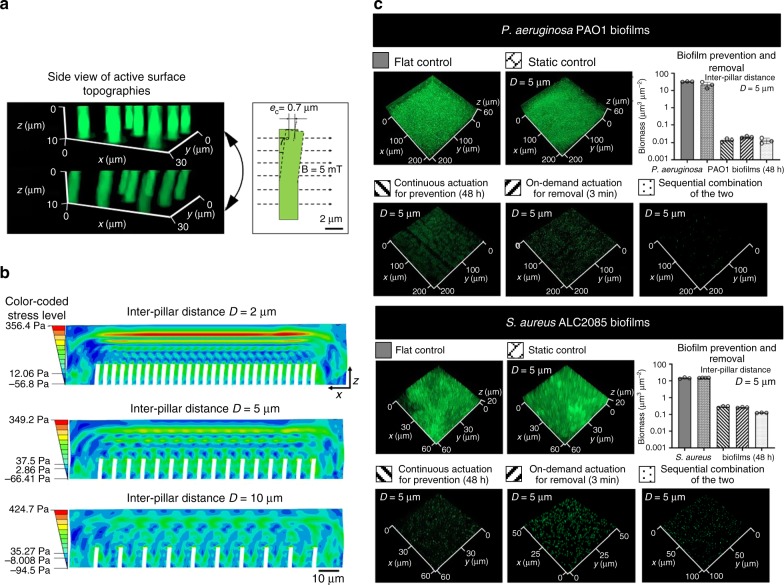
Optimization of the pillar design. **a** Bending of PDMS pillars with a height of 10 µm, diameter of 2 µm, inter-pillar distance of 5 µm, and Young’s modulus of 2.1 MPa in response to a 5 mT magnetic field. The pillars appeared green in fluorescence microscopy after being labeled with Acridine Orange. **b** Stress propagation in viscoelastic biofilms generated by the on-demand beating of active pillars with different inter-pillar distances (*D* = 2, 5, and 10 µm). Active pillars (in white color) were actuated using a 5 mT magnetic field. **c** Representative fluorescence images of biofilms on flat controls, static controls, and PDMS surfaces with active surface topographies. The pillars were 10 µm tall with a diameter of 2 µm and inter-pillar distance of 5 µm. Active surface topographies were operated in three modes, including continuous beating for biofilm prevention, on-demand removal of mature biofilms (only actuated for 3 min after 48 h of biofilm growth), and a sequential combination of these two treatments. Biofilm cells were labeled with STYO^®^9. The biomass was quantified using COMSTAT^59^. The samples in the bar graph are indicated with the pattern labels above the corresponding fluorescence images. Each condition was tested with at least three biological replicates (error bar = standard deviation; *n* = 3–4), and five random images were taken from each sample. Source data of Fig. 3c are provided in the Source Data file.

To test this hypothesis, we modeled the on-demand beating of active pillars with varying inter-pillar distance (2, 5, and 10 µm) in 29 µm thick biofilms (the average thickness of 48 h *P. aeruginosa* PAO1 and *S. aureus* ALC2085 biofilms in this study) (Fig. 3a, b). The results of simulation with the CPS4R element revealed that the pillars instantaneously reach the maximum displacement (in less than 0.02 ms). Using a model of cantilever beam deflection^41^, the maximum displacement of each pillar in a 5 mT electromagnetic field was estimated to be 0.7 µm (details described in Supplementary Methods 1.1), which is the same as the experimental observation using microscopy (Fig. 3a). Such deformation of pillars generated stress at the tips of the pillars and a stress wave propagating from the biofilm-pillar interface to the top of a viscoelastic biofilm (Fig. 3b). The stress wave was reflected from the free surfaces and kept propagating inside the biofilm for 10 ms. During a 3-min on-demand actuation, the stress at the tips could break the interaction between biofilms and pillars, and the stress propagation through biofilm could disrupt the 3D biofilm structure. Which impact occurs first and/or dominates depends on the biofilm structure and related material properties. We observed continuous shedding of individual UPEC biofilm cells during actuation (Supplementary Fig. 7 and Supplementary Movie 1), indicating the disruption of 3D biofilm structure rather than peeling off the entire biofilm. To understand the effects on viscoelastic biofilms, we stained the eDNA in the matrix of 48 h *P. aeruginosa* PAO1 biofilms. By tracking the movement of a reference point during 3-min on-demand actuation, we also observed stress propagation due to pillar movement (Supplementary Fig. 8 and Supplementary Movie 2). This propagation stretched the biofilm matrix, further supporting that the removal of *P. aeruginosa* biofilms during 3-min on-demand actuation was due to structural disruption. Additional research is needed to further understand the mechanisms behind active topography triggered biofilm failure, which is part of our ongoing work.

Among all the three inter-pillar distances compared (*D* = 2, 5, or 10 µm), simulation results showed that the stress on the top layer of the viscoelastic *P. aeruginosa* PAO1 biofilm was the highest when *D* was 2 µm (356.4 Pa; red color in Fig. 3b) and the lowest when *D* was 10 µm (165.1 Pa; green color in Fig. 3b). This is consistent with the experimental results showing that on-demand actuation of pillars with an inter-pillar distance of 10 µm was the least effective design in disrupting *S. aureus* biofilm (Supplementary Table 2; Supplementary Fig. 9). Active pillars with the spacing of 2 and 5 µm generated similar levels of stress in biofilm removal based on the simulation results. Experimentally, however, *P. aeruginosa* PAO1 biofilm cells were stuck between pillars when *D* was 2 µm (Supplementary Fig. 10). In comparison, surfaces with 5 µm spacing exhibited optimal antifouling activities against both *P. aeruginosa* PAO1 and *S. aureus* ALC2085 (Supplementary Tables 1, 2 and Fig. 3c). This optimal inter-pillar distance (*D* = 5 µm) was further corroborated by the enhanced antifouling effects against 48 h UPEC ATCC53505 biofilm formation. Specifically, there were 0.002 ± 0.0003 and 0.002 ± 0.002 µm^3^ µm^−2^ UPEC biomass after continuous or on-demand actuation, respectively. Both had a 3.7-log reduction (*p* = 0.0057 and 0.0003, respectively) in biomass compared to their flat control (9.2 ± 3.0 µm^3^ µm^−2^; Supplementary Fig. 11). The SEM images confirmed effective biofilm removal by 3 min on-demand actuation of the optimized active topography (Supplementary Fig. 7). Compared to those pillars with *D* of 2 µm, there was 1.0 and 0.8-log more reduction of biofilm cells with continuous or on-demand actuation, respectively (Supplementary Table 3; Supplementary Fig. 11). Hence, active pillars with 5 µm inter-pillar distance was used throughout the rest of this study.

### Detached cells were sensitized to bactericidal antibiotics

A hallmark characteristic of mature biofilms is the high-level antibiotic tolerance of biofilm cells (e.g., up to 1,000-times higher than planktonic cells^4,5,7^). This is attributed to multiple factors, especially the protection of biofilm matrix and reduced cell metabolism and growth^5^. Recently, we reported that biofilm detachment triggered by shape recovery of a shape memory polymer (SMP) increased the susceptibility of *P. aeruginosa* PAO1 cells to tobramycin by 3 logs^42^. Given the strong activities in biofilm removal, we expected that active topographies described above would have similar effects. Specifically, we hypothesized that biofilm dispersion by active topography would alter the physiological stage of biofilm cells, and thus their antibiotic susceptibility. To test this hypothesis, we evaluated the antibiotic susceptibility of biofilm cells detached during on-demand actuation of pillars; and compared it with that of cells remained on the surface, cells dispersed by bead beating, and those in intact biofilms (treated without cell detachment).

**Fig. 4:**
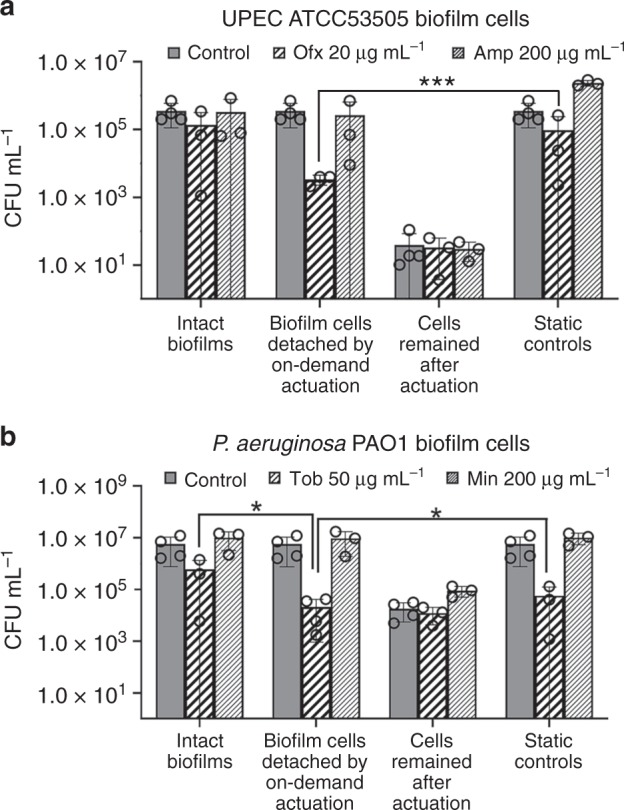
On-demand actuation sensitized biofilm cells to bactericidal antibiotics. **a**, **b** Antibiotic susceptibility of 48 h UPEC ATCC53505 (**a**) and *P. aeruginosa* PAO1 (**b**) biofilm cells after treatment for 1 h in 0.85% NaCl at 37 °C. UPEC ATCC53505 biofilm cells were treated with 20 µg mL^−1^ Ofx (bactericidal) and 200 µg mL^−1^ Amp (bacteriostatic). *P. aeruginosa* PAO1 cells were treated with 50 µg mL^−1^ Tob (bactericidal) and 200 µg mL^−1^ Min (bacteriostatic). For the samples that went through on-demand actuation, the remaining biofilm cells were detached by bead beating (25 Hz bead beating for 30 s). All of the dispersed cells also went through the same bead beating step to eliminate artifacts before they were treated with an antibiotic. The biofilm cells dispersed by beat beating and then treated with an antibiotic are referred to as Static controls, and biofilms treated directly with an antibiotic without dispersion are referred to as Intact biofilms. Each condition was tested with at least three biological replicates (error bar = standard deviation; *n* = 3–4; ***p < 0.0001 and *p < 0.05, two-way ANOVA adjusted by Tukey test). Source data of Fig. 4 are provided in the Source data file.

The 3-min on-demand actuation was found to sensitize UPEC ATCC53505 biofilm cells to ofloxacin (Ofx, a bactericidal antibiotic). Compared to the cells detached by bead beating as a control method of mechanical biofilm removal, 1 h of treatment with 20 µg mL^−1^ Ofx caused 1.5-log more killing of cells detached by on-demand actuation (*p* < 0.0001; *n* = 3; Fig. 4a). Bead beating itself has no effects on cell viability^42^, and biofilm cells dispersed by on-demand actuation also went through bead beating to avoid any confounding effects. We also verified that there was no detectable amount of MNPs in the solution after bead beating under the same condition of the viability assay (25 Hz for 30 s). Since the detection limit in our system is 3.2 ng and 1 µg MNPs were loaded in the pillars per sample, at least 99.7% of the beads were retained during the beating. To more directly evaluate the possible effects of MNPs (if any is released), we repeated the experiment of Ofx (20 µg mL^−1^) treatment of exponential phase planktonic UPEC cells, but with the addition of 1 µg MNPs and MNP-free pillars (to simulate the condition when all MPNs are released). No difference in Ofx killing was observed between the two conditions (*p* = 0.20; *n* = 3). Bead beating with 1 µg MNPs at 25 Hz for 30 s also showed no effects on the viability of exponential phase cells (*p* = 0.09; *n* = 3). These results further support that the enhanced killing by Ofx was due to active topography.

Antibiotic susceptibility of UPEC biofilm cells detached by bead beating was similar to that of cells in the intact biofilms (Fig. 4a; *p* > 0.05). This suggests that the increase in Ofx susceptibility by active topography was not due to the disruption of biofilm structure alone. We speculate that active topography may change the physiology of detached cells to a more active stage, leading to increased antibiotic susceptibility, as we observed for the cells detached by topographic changes of SMP^42^. To understand if active topography causes such effects, we conducted a qPCR experiment to track the changes in the expression of *rrnB* genes (*rrsB*, *rrlB*, and *rrnB* P1) right after the 3-min on-demand actuation, and after 7 and 30 min further incubation in 0.85% NaCl solution. These genes encode 16s rRNA, 23s rRNA, and the space-1 region, respectively, and have been used to track the expression level of *rrnB* operon to monitor cellular activities^42,43^. The results showed that the expression of these three genes increased over time in UPEC biofilm cells detached by on-demand actuation (*p* < 0.0004, two-way ANOVA adjusted by Tukey test), but not in the cells that remained on the surface and detached by bead beating (*p* > 0.05, two-way ANOVA adjusted by Tukey test; Supplementary Fig. 12). The expression level of *rrnB* operon positively correlates with the growth rate of bacterial cells^43^. Thus, the induction of *rrnB* genes in cells detached by active topography can help explain the increase in antibiotic susceptibility of these detached cells.

In comparison, a bacteriostatic antibiotic, ampicillin (Amp), did not show similar effects (*p* > 0.05, two-way ANOVA adjusted by Tukey test; Fig. 4a). It is worth noticing that 3-min on-demand actuation reduced the number of 48 h UPEC ATCC53505 biofilm cells by 4.0 logs, leaving only 3.3 cells mm^−2^ on the surface (Fig. 4a). This result is consistent with the 3.7-log reduction in biomass compared to the static control, as observed using a fluorescence microscope (Supplementary Fig. 11; Supplementary Table 3). On-demand actuation also sensitized mature *P. aeruginosa* PAO1 biofilms to bactericidal tobramycin (Tob) compared to cells dispersed by bead beating and those inside intact biofilms (with 0.4 and 1.4-log more killing, respectively; *p* < 0.05, two-way ANOVA adjusted by Tukey test), but not bacteriostatic minocycline (Min) (Fig. 4b). These results indicate that the increase in antibiotic susceptibility of cells detached by active topography is species non-specific.

### Safety to mammalian cells

To evaluate the clinical potential of this design, we further tested its safety to mammalian cells. Because PDMS has been widely used in medical implants^37^, the material itself is not a concern. Actuation of the PDMS pillars in our design is powered by an electromagnetic field produced from solenoid coils. The copper wires are insulated, and therefore, the electric current is not a safety concern. We thus focused our study on evaluating if there is heat production by the electric current (for generating the external magnetic field) and the possible leaching of MNPs from the tips of pillars.

**Fig. 5:**
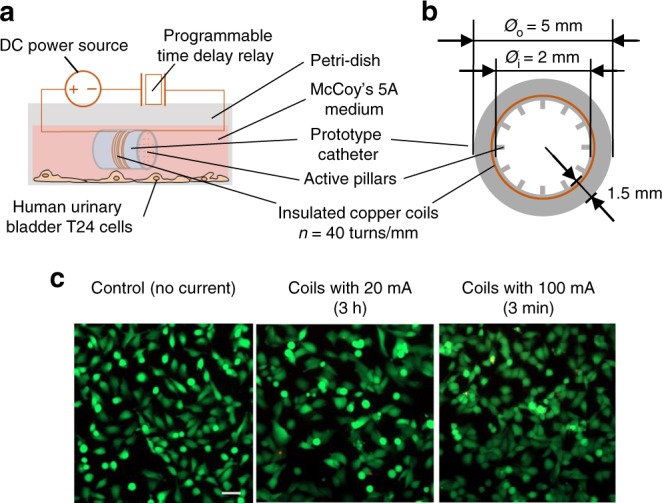
Biocompatibility of active topographies. **a**, **b** Schematic of the experimental setup (**a**) and a cross-sectional view of the catheter prototype (**b**). **c** Effects of insulated copper coils on transformed human urinary bladder T24 cells without or with actuation (1 mT for 3 h or 5 mT for 3 min; Bar = 50 µm). Each condition was tested with three biological replicates (*n* = 3), and five random images were taken from each sample.

Both modeling (details shown in Supplementary Methods 1.2) and experimental results indicate that heat generation is negligible and will not cause harm to mammalian cells. With a coil density of 40 turns mm^−1^ and oscillation frequency of 10 Hz, the electric current-induced temperature change at the polymer surface is estimated to be 2.1 × 10^−8^ °C per coil after 3 h of continuous actuation (1 mT induced with a 40 mA current) and 7.9 × 10^−9^ °C per coil after 3 min on-demand actuation (5 mT induced with a 200 mA current). These temperature changes are negligible. The local heat generation at the pillar tip is also negligible (details shown in Supplementary Methods 1.3). To corroborate the modeling results, we cultured human urinary bladder T24 (ATCC® HTB-4) cells under the same actuation conditions (1 mT for 3 h or 5 mT for 3 min) with a prototype catheter carrying active pillars on the inner wall (Fig. 5a, b). The results revealed no change in cell viability (Fig. 5a–c). Furthermore, copper coils are widely used in the medical field, such as intrauterine device (IUD) for birth control; and the level of the electromagnetic field used in this design (1-5 mT) is significantly lower than the exposure limit recommended by the World Health Organization (40 mT)^44,45^.

Another factor to consider for the design is the biocompatibility of MNPs that are embedded at the tips of PDMS pillars. Magnetic iron oxide nanoparticles have been widely used in biological and biomedical applications with good biocompatibility^46^. In this study, only 7.6 × 10^−9^ mg MNPs were loaded in each pillar, a low amount that is expected to be safe. To further understand if there is any leak of MNPs during actuation, we monitored MNP release during continuous actuation of active pillars at 37 °C in phosphate-buffered saline (PBS) solutions with varying pH (6, 7, or 7.4 within the normal pH range of human urine). Using a ferrozine assay^47^, we did not detect any MNPs in PBS solutions after 48 h of actuation. Since the detection limit of this assays is 3.2 ng based on our standard curve, at least 99.7% of the MNPs remained in the pillars during actuation.

### Long-term antifouling activities of the prototype catheter

**Fig. 6:**
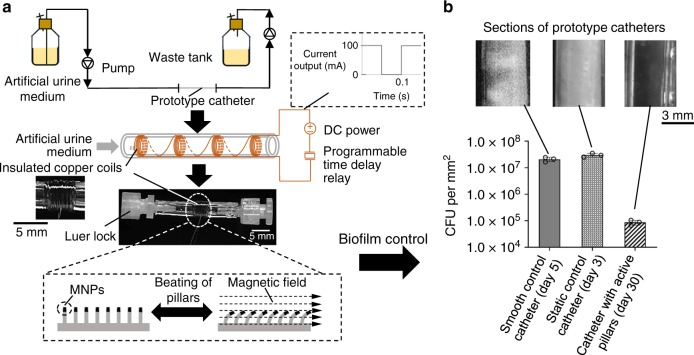
Long-term test of the prototype catheters. **a** Schematic of the flow cell system for testing long-term antifouling activities of the prototype catheters. A zoom-in view shows the coil section. **b** Antifouling activities of the prototype catheters. The bar graph shows the CFU per unit interior lumen area. The representative images show the complete blockage (visible white substance) of static control (on day 3) and flat control (on day 5) catheters, while the prototype catheters remained clear on day 30. The coils for generating the magnetic field were removed before imaging in Fig. 6b. The catheters were incubated at 37 °C with a continuous flow of artificial urine medium at 10 mL h^−1^. Each condition was tested with three biological replicates (error bar = standard deviation; *n* = 3). Source data of Fig. 6b are provided in the Source data file.

Biofilm formation in urinary catheters is the primary cause of CAUTI^3^. The strong antifouling effects of active topography bring a unique opportunity for engineering self-cleaning medical devices such as catheters. To understand if continuous actuation can provide long-term biofilm control, we constructed a prototype catheter based on the optimized design of active pillars with a height of 10 µm, diameter of 2 µm, and inter-pillar distance of 5 µm (Fig. 6a). The magnetic field was generated using an insulated copper coil, and the beating frequency was controlled by a programmable time delay relay. With 10 mL h^−1^ flow of artificial urine medium, UPEC ATCC53505 formed thick biofilms and completely blocked the static control catheters (with static pillars) in 3 days (Fig. 6b) with a biofilm cell density of (3.0 ± 0.7) × 10^7^ cells per unit interior lumen area (mm^2^). Similar blockage of the smooth control catheter (without pillars; Fig. 6) happened within 5 days after inoculation with a biofilm cell density of (2.0 ± 0.6) × 10^7^ cells mm^−2^ (Fig. 6). In comparison, active topography (driven by a 1 mT magnetic field) kept the prototype catheter clear even 30 days after inoculation when the experiment was terminated as the set goal was met (30 days of biofilm prevention). Consistent with the strong antifouling activities to remain clear, the prototype catheters with active topography also had much fewer cells associated [(8.6 ± 2.6) × 10^4^ cells mm^−2^)] on day 30 than the static control on day 3 [(3.0 ± 0.7) × 10^7^ cells mm^−2^] and flat control on day 5 [(2.0 ± 0.6) × 10^7^ cells mm^−2^].

## Discussion

The severe problems associated with drug-resistant biofilm infections stimulated increasing interest in developing bacterial control strategies that do not require antimicrobial agents^26^. A number of static topographies have been shown to inhibit biofilm formation for varying duration^48–51^. However, bacteria can eventually overcome unfavorable topographies through growth and biofilm matrix production^29^. We recently reported biofilm control using dynamic topography via an on-demand change in surface topography of SMP^52^. This method achieved up to 3-log removal of mature biofilms of both bacteria and fungi; however, shape recovery can only occur a limited number of times, and in most SMP systems, only once. Effective systems for long-term fouling control are still missing.

In this study, we developed a fouling control strategy based on active topography that is programmable and applicable to a variety of polymeric materials. This is achieved by remotely controlling the beating of rationally designed micron-size pillars using an external electromagnetic field. This system is programmable, and thus, has the potential to be integrated with other sensors to engineer smart medical devices that can adjust pillar beating frequency and force for microbial control. Most biofilm control technologies developed to date aim to prevent microbial attachment (e.g., static topographies)^23–26^, with few studies on the removal of mature biofilms (dynamic strategies)^52–55^; and no method is equally effective in both. Here we demonstrate that active topography developed in this study can achieve both controls with a tunable force level generated by beating pillars.

The optimal inter-pillar distance in our design was found to be 5 µm. According to Bottier et al.^36,38^, when motile cilia beat in a liquid medium, the synchronized movement of epithelial cilia [cilia length (*H*) = 10 µm and beating frequency (*ω*) = 10 Hz] could generate a 5 µm wide high-velocity areas close to the epithelial cell wall. In this study, active pillars were embedded in mature biofilms formed in static growth medium with mechanical properties similar to hydrogels (e.g., Young’s modulus of *P. aeruginosa* PAO1 biofilm formed in LB medium is 2.0 kPa)^56^. Using numerical simulation with CPS4R element, the beating of active pillars with a modulus of 2.1 MPa^57^ was found to generate strong local stress at the tips of pillars and a stress wave propagating from the biofilm-pillar interface to the top surface of the biofilm. The maximum stress can reach 356.4 and 245.3 Pa when the inter-pillar distances were 2 and 5 µm, respectively (Fig. 3b). This is strong enough to disrupt the 3D *P. aeruginosa* PAO1 biofilm structures in 3 min, according to the relaxation time reported by Rozenbaum et al.^56^. This stress was also strong enough to disrupt *S. aureus* ALC2085 biofilms, which were the most challenging ones in this study. Although our simulation showed the strongest stress on biofilm structure when the pillars were 2 µm apart, the surface with 5 µm inter-pillar distance showed the best activities in both biofilm prevention and removal. This is probably because the close inter-pillar distance (2 µm) caused some cells to be trapped, and thus, not removed by gentle wash after pillar actuation (Supplementary Fig. 10). To further understand the effects of pillar spacing, we also tested early-stage biofilm formation of the three species at 3 and 6 h after inoculation. The results indicate that spacing of 5 µm is more effective in preventing initial attachment and early biofilm development than the spacing of 2 µm, consistent with the 48 h biofilm results (Supplementary Figs. 2–4). It is worth noticing that it only took 0.02 ms for the pillars to reach their maximum displacement in an electromagnetic field of 5 mT, which is much shorter than the on/off time during each oscillation (0.05 s). Hence, there is a large room for further optimization. Apparently, the mechanical properties of biofilms formed by different species can affect the efficiency of active pillars, which deserves future study. If needed, the force can be increased by adjusting the magnetic field strength and/or beating frequency.

In addition to removing biofilm cells from the surface, active topography also rendered the detached cells more susceptible to bactericidal antibiotics. For instance, UPEC ATCC53505 biofilm cells dispersed by on-demand actuation was more sensitive (with 1.5-log more killing) to 20 µg mL^−1^ Ofx compared to biofilm cells detached by bead beading, and similar to that of planktonic cells in exponential phase (with 99.3 ± 0.6% killing). Consistently, we observed an increase in the expression of *rrnB* genes in cells dispersed by active pillars, compared to the cells remaining on the surface and biofilm cells dispersed by bead beating. Recently, we reported that SMP-based dynamic topography induced biofilm dispersion and sensitized *P. aeruginosa* PAO1 biofilms to bactericidal antibiotics^42^. Such biofilm dispersion was found to increase the intracellular level of ATP and expression of the *rrnB* gene^42^. Thus, active surface systems represent a strategy with added benefits to eradicate biofilm cells and may help control chronic infections through synergy with conventional antibiotics. The long-term antifouling activities of the prototype catheter and the safety of this system to mammalian cells demonstrated the potential of this design for controlling CAUTI. In addition to the significant reduction of bacterial load, the prototype catheter was found effective in preventing biofilm blockage, which plays a major role in CAUTI^3^.

In summary, we have developed an antifouling strategy based on active topography that can achieve long-term biofilm control. The antifouling effects of this active surface system are based on the programmable movement of micron-scale pillars in response to an external electromagnetic field. This approach can both prevent microbial adhesion and remove mature biofilms. Because the pillars are fabricated using lithography, this strategy can be readily applied to a wide range of materials, including those in medical devices such as urinary catheters. Further studies using animal models will help validate the clinical potential of this technology, which is part of our future work.

## Methods

### Strains and growth media

*P. aeruginosa* PAO1, UPEC ATCC53505, and *S. aureus* ALC2085 were used as the model microorganisms in this study. Bacterial cells were first grown in Lysogeny Broth (LB) overnight at 37 °C with shaking at 200 rpm. Overnight cultures were used to inoculate biofilm cultures with an optical density at 600 nm (OD_600_) of 0.05. Biofilm samples were harvested at 48 h after inoculation to test the antifouling effects of active PDMS pillars.

### Synthesis of superparamagnetic Fe_3_O_4_ nanoparticles (MNPs)

The protocol described by Myrovali et al.^58^ was followed with minor modifications. In detail, MNPs was synthesized by mixing FeCl_2_ powder (4.225 g, Sigma-Aldrich, St. Louis, MO, USA) with FeCl_3_ powder (10.8 g) in distilled water (200 mL) to obtain an iron concentration of 0.5 M with the Fe^2+^ to Fe^3+^ ratio of 1:2 and heated up to 80 °C in a water bath under continuous stirring^58^. During stirring, 0.1 M NaOH solution was rapidly added to adjust pH to 10.5. The suspension turned black, indicating the formation of nanoparticles. Then the system was aged with slow stirring at 80 °C for another 2 h^58^. After being cooled down slowly to room temperature, the MNPs were washed with deionized (DI) water three times with centrifugation at 2,300 × *g* for 5 min and gentle dispersion. MNPs were dried at 60 °C for 2 h before use.

### Determining the size distribution of MNPs

To visualize the MNPs with TEM, we diluted and suspended the MNPs to 1 mg mL^−1^ in DI water. A drop of MNPs suspension (10 µL) was deposited onto the formvar-carbon coated TEM grid (300 mesh gold, Electron Microscopy Sciences, Hatfield, PA, USA). Then, the sample was dried under vacuum for 24 h and observed using a TEM (JEOL JSM-2000EX, Tokyo, Japan) operated at 200 kV (a representative image shown in Fig. 1a).

### Surface preparation

To introduce active surface topographies onto PDMS surfaces, silicon wafers with complementary patterns were fabricated at the Cornell Nanoscale Science & Technology Facility (CNF; Cornell University, Ithaca, NY, USA) by following the protocol described by Hou et al.^18^. In detail, the configuration of the pillars was designed using the software L-Edit (Tanner Research, Monrovia, CA, USA) to create circular shapes with a diameter of 2 µm with varying inter-pillar distance (2, 5, 10, or 15 µm). This design was first written onto a photomask using a photomask writer (Heidelberg Mask Writer—DWL2000) and then transferred onto silicon wafers coated with photoresist S1813 using ABM contact aligner. The circular holes on silicon wafers were etched to obtain approximately 10 µm deep features using a PlasmaTherm Unaxis 770 Deep Silicon Etcher. To minimize PDMS residues in each round of soft lithography, silicon wafers were coated with (tridecafluoro-1,1,2,2,-tetrahydrooctyl)trichlorosilane (FOTS) prior to use as templates.

To load MNPs in PDMS pillars, MNPs were dispersed in a mixture of silicone elastomer [base: curing agent = 10:1 (wt/wt); Sylgard^®^ 184 elastomer kit, ThermoFisher Scientific, Waltham, MA, USA] with a weight ratio of 1:4 (wt/wt) with gentle mixing. Then PDMS surfaces with evenly distributed MNPs prepared as described above were applied onto the silicon wafers with complementary patterns and vacuumed for 1 h with a 1 T magnet (Master Magnetics Inc., Castle Rock, CO, USA) placed under the wafer to force the MNPs to the bottom of the wells. After vacuuming, the excessive mixture of PDMS and MNPs were removed from the silicon wafer, and clean PDMS was applied onto the silicon wafer to form the base. PDMS was polymerized at 60 °C for 24 h, and the surface was peeled off gently after the magnets were removed (Fig. 1a). PDMS surfaces with active pillars were stored at room temperature until the following test. Flat PDMS surfaces were also prepared as controls.

To validate that the MNPs settled through silicone elastomer mixture, a separate test was run to visualize this process in transparent cuvettes [12.5 mm (width) × 12.5 mm (length) × 45 mm (height); Fig. 1b]. The cuvettes were filled with clean silicone elastomer mixtures until the surface of the mixtures was 10 mm from the bottom. Then, MNPs dispersed in the mixture of silicon elastomer were added at the top of the clean silicon elastomer mixture. To drive the movement of MNPs in silicone elastomer, a 1 T magnet was positioned at the bottom of the cuvette as described above. Gravity controls were included without magnets. By tracking the movement of MNPs in the mixture of silicone elastomer in real-time, the settlement of MNPs was monitored by plotting the distance from the bottom of cuvettes (*y*-axis) versus the settlement time of MPNs (*x*-axis).

To further map the distribution of MNPs in pillars, we repeated the pillar fabrication with Alexa Fluor 488 conjugated Dextran CLIO MNPs (5 mg mL^−1^; Luna Nanotech, Toronto, ON, Canada). We took 3D images of the active pillars using fluorescence microscopy (Axio Imager M1 fluorescence microscope, Carl Zeiss Inc., Berlin, Germany) with a *z*-axis interval of 1 µm. Excitation at 498 nm and emission at 519 nm were used to detect the green fluorescence signal from the fluorescently tagged MNPs, and brightfield was used to identify the profile of the pillars. The 3D fluorescence images were deconvoluted using Zen Blue (Carl Zeiss Inc., Berlin, Germany) to remove background noise (Fig. 1c). To track the movement of pillars in a magnetic field using fluorescence microscopy, we labeled the pillars with Acridine Orange (ThermoFisher Scientific, Waltham, MA, USA), and took 3D fluorescence images of the pillars during actuation using an excitation wavelength of 500 nm and emission wavelength of 526 nm (green fluorescence in Fig. 3a).

The distribution of MNPs in pillars was also confirmed using SEM-EDS. Clean and dry PDMS surfaces with pillars (no bacterial cells) were sputter-coated in a platinum sputter (Edwards S150A, Edwards, Burgess Hill, UK) under 30 mV with 75 s deposition time, and then imaged using JEOL JSM-IT 100LA SEM (JEOL Ltd., Tokyo, Japan). Besides taking the SEM images, the EDS technique was used to detect the signals emitted from the Fe atoms to locate the MNPs.

### Antifouling assay

To test the performance of active surface topographies in biofilm prevention, PDMS surfaces with fabricated pillars were sterilized in a clean petri dish first using UV for 1 h with the patterned side facing up. Then, each surface was transferred into a 15 mL falcon tube placed horizontally with the bottom of the PDMS surface (non-patterned side) attached to the inner wall of the Falcon tube. Meanwhile, an insulated copper coil (200 turns in 5 mm) was positioned at the same location to wrap the outer wall of the Falcon tube. The copper coil was made with a copper wire (with a diameter of 0.127 mm). Bacterial cells were inoculated in LB medium (13 mL) in the tube for biofilm growth with an initial OD_600_ of 0.05. The surface was actuated for 3, 6, or 48 h continuously during incubation at 37 °C by turning a 40 mA DC on and off at a frequency of 10 Hz using a programmable (multi-function) time delay relay. Biofilms formed under the same conditions on PDMS surfaces with the same pillars but without actuation were used as controls. To sample biofilms, PDMS surfaces with or without actuation were gently washed three times with 0.85% (wt/vol) NaCl solution.

The biomass of attached cells was quantified by labeling the cells with SYTO^®^ 9 and imaged using an Axio Imager M1 fluorescence microscope (Carl Zeiss Inc., Berlin, Germany). For each inter-pillar distance and the flat PDMS surfaces, 3D fluorescence images were taken from five randomly selected positions on each PDMS surface using an excitation wavelength 488 nm and emission wavelength of 506 nm with a *z*-axis interval of 1 µm. No autofluorescence was observed from PDMS pillars with SYTO^®^ 9 labeling. The 3D fluorescence images were deconvoluted before biomass analysis using MATLAB 2015a (Mathworks, Natick, MA, USA) based COMSTAT^59^. The results were compared with those of PDMS surfaces with static surface topographies and flat PDMS surfaces (defined as static and flat controls throughout this study).

To test the removal of mature biofilms, 48 h biofilms were formed on PDMS surfaces without pillar actuation first in Petri dishes at 37 °C with an inoculation OD_600_ of 0.05. Before on-demand actuation, PDMS surfaces were washed three times with 0.85% NaCl solution first. Then, a 5 mT electromagnetic field was applied for 3 min. After the on-demand biofilm removal, PDMS surfaces were washed three times with clean 0.85% NaCl solution. Then the remaining biofilm cells were labeled with SYTO^®^9 and imaged as described above. To study the effects of 3-min on-demand actuation on UPEC ATCC53505 and *P. aeruginosa* PAO1 biofilms, time-lapse movies (with a time-interval of 4.28 and 0.26 s between frames, respectively) of biofilms labeled with SYTO^®^9 was taken during on-demand actuation (Supplementary Movies 1, 2). The movement of a reference point in both *x* and *y*-directions was analyzed for *P. aeruginosa* PAO1 biofilms (Supplementary Fig. 8 and Supplementary Movie 2).

The sequential combination of the two actuation modes (continuous and on-demand actuation) was conducted by increasing the strength of the magnetic field to 5 mT for 3 min at the end of a 48 h continuous actuation at 1 mT. The biomass of biofilms with and without the sequential actuation was quantified in the same way as described above for continuous or on-demand actuation.

### Biofilm imaging using SEM

UPEC ATCC53505 biofilms (48 h) on PDMS surfaces with static pillars were formed as described above for on-demand actuation. After biofilm formation, mature biofilms were processed with or without 3 min on-demand actuation as described above. Then, PDMS surfaces were transferred into PBS with 2.5% glutaraldehyde and soaked for at least 30 min to fix the biological structures. Glutaraldehyde solutions with PDMS surfaces were stored at 4 °C until sample preparation for SEM. Before SEM analysis, PDMS surfaces were dehydrated using 15, 30, 50, 70, 95, and 100% ethanol for 15 min each. The 100% ethanol treatment step was repeated three times. Samples were then imaged using JEOL JSM-IT 100LA (JEOL Ltd., Tokyo, Japan) after they were sputter-coated in a platinum sputter (Edwards S150A, Edwards, Burgess Hill, UK) under 30 mV with 75 s deposition time.

### Simulation of stress propagation in mature biofilms

We developed a two-dimensional (2D) finite-element model of dynamic deformation of the hybrid biofilm and pillar structures. As shown in Supplementary Fig. 13, an array of PDMS pillars (10 µm tall and 2 µm in diameter) were embedded in a biofilm strip with length 135 µm and height 29 µm (average thickness of *P. aeruginosa* and *S. aureus* biofilms in this study). The distance between pillars varied from 2 to 10 µm. The deformation of the biofilm and pillars were assumed to be under plane-stress condition, as a simplified model of three-dimensional (3D) biofilm slab with a 2D array of pillars. The instantaneous elastic properties of biofilm and PDMS pillar were modeled as the neo-Hookean material, whose strain energy density can be expressed as Eq. (1):

1 $$U = \frac{1}{2}\mu \left( {I_1J^{ - \frac{2}{3}} - 3} \right) + \frac{1}{2}K\left( {J - 1} \right)^2,$$

where *U* is the strain energy density function; *μ* is the shear modulus; *K* is the bulk modulus; *I*_1_ represents the first invariant of the right Cauchy-Green strain tensor ***C*** = ***F***^T^***F*** (***F*** is the deformation gradient tensor); and *J* is the volumetric strain. The first Piola-Kirchhoff stress tensor ***P*** can be expressed as $\boldsymbol{P}= \frac{{\partial U}}{{\partial {\boldsymbol{F}}}}$. The viscoelastic properties of the biofilms are modeled as a generalized Maxwell model with four different relaxation time and moduli:

2 $$E\left( t \right) = E_0 + E_1e^{ - \frac{t}{{\tau _1}}} + E_2e^{ - \frac{t}{{\tau _2}}} + E_3e^{ - \frac{t}{{\tau _3}}},$$

where *E*_*i*_ and *τ*_*i*_ represent the elastic modulus and the relaxation time, respectively.

All numerical simulations were carried out with ABAQUS/Standard. The biofilm and pillars were modeled with CPS4R element. The Young’s modulus of the pillars was taken as 2.1 MPa. We run FEM simulations to fit the parameters with the experimental data of creep test of *P. aeruginosa* biofilms grown in LB medium^56^, which gives *E*_0_ = 0.02 kPa, *E*_1_ = 0.06 kPa, *τ*_1_ = 0.02 s, *E*_2_ = 1.8 kPa, *τ*_2_ = 0.2 s, and *E*_3_ = 0.12 kPa, *τ*_3_ = 3.0 s (Supplementary Fig. 14). The viscoelastic properties were implemented in ABAQUS through the Prony series. To approximate the incompressibility, the Poisson’s ratio of the two materials were set to be 0.475. The densities of the two materials were equal to 1000 kg m^−3^. To simulate the active force of the magnetic particles at the tip of the PDMS pillars, we applied a distributed force at the top surface of the pillar with a magnitude of 0.00225 N mm^−2^. This generated a maximum 0.7 µm displacement in the pillars, matching exactly with the experimental measurement. We simulated two cycles of the loading (turning on the magnetic field) and unloading (turning off the magnetic field) process. Within each cycle, the forces were applied to the pillars at the starting point of the simulation and kept the same magnitude for 0.05 s and then removed to let the system relax for another 0.05 s (Supplementary Fig. 13).

### Antibiotic susceptibility test

To test the effects of on-demand actuation on antibiotic susceptibility of mature biofilm cells, we treated biofilm cells with both bactericidal and bacteriostatic antibiotics. Specifically, UPEC ATCC53505 biofilm cells were treated with 20 µg mL^−1^ Ofx and 200 µg mL^−1^ Amp; and *P. aeruginosa* PAO1 biofilm cells were treated with 50 µg mL^−1^ Tob and 200 µg mL^−1^ Min. In detail, after 48 h biofilm formation on PDMS surfaces with static surface topographies, the PDMS coupons were washed three times with 0.85% NaCl solution before antibiotic treatment. Antibiotic treatment of intact biofilms was conducted by directly transferring the washed biofilms into 0.85% NaCl solution with or without antibiotics and incubating for 1 h at 37 °C without shaking. Then, they were transferred into clean 0.85% NaCl solution with 0.1 g of 0.1 mm zirconia/silica beads after three times of gentle wash with clean 0.85% NaCl solution. Biofilm cells were harvested after 30 s of bead beating at 25 Hz. The static controls were transferred to 0.85% NaCl solution with 0.1 g of 0.1 mm zirconia/silica beads (BioSpec Products, Inc., Bartlesville, OK, USA) and beat at 25 Hz for 3 min to collect cells. Cells dispersed by active topography also went through bead beading to eliminate any artifacts. The collected cells were then suspended in clean 0.85% NaCl solution with or without an antibiotic and incubated for 1 h at 37 °C. Cell viability was determined using the drop plate method after washing three times with clean 0.85% NaCl solution as described by Naghili et al.^60^. In detail, biofilm cells in 200 µL 0.85% NaCl solution were diluted up to eight times with a dilution factor of 10 by transferring 20 µL bacterial solution into 180 µL clean 0.85% NaCl in a 96-well plate. After mixing, 10 µL of each 200 µL bacterial suspension was dropped onto an LB agar plate. LB agar plates were incubated at 37 °C overnight after the droplets were air-dried, and the number of colony-forming units (CFU) was counted. The PDMS surfaces with active topographies went through the same process. The only difference is that the active surfaces were exposed to a 5 mT electromagnetic field during the 3 min incubation to trigger biofilm dispersion.

### qPCR analysis

After 48 h UPEC ATCC53505 biofilm formation on PDMS surfaces with static or active pillars, biofilm cells were detached by 3 min on-demand actuation or bead beating (control) as described above. The remaining UPEC biofilm cells were also removed by bead beating, and the biofilm cells  dispersed by on-demand actuation were subjected to bead beating as well to eliminate any confounding effect. The cells were harvested at 0, 7, or 30 min after on-demand actuation or bead beating. RNA was isolated by following the protocol of RNeasy Mini Kit (Qiagen, Hilden, Germany). To conduct qPCR, the RNA was reverse transcribed into cDNA using the iScript cDNA Synthesis Kit (Bio-Rad Laboratories, Hercules, CA, USA). Three genes, including *rrsB* (16s rRNA), *rrlB* (23s rRNA), and *rrnB* P1 (spacer-1 region), were amplified using the primers listed in Supplementary Table 4. The gene *rrsA* was used as the housekeeping gene^61^. The qPCR was conducted with denaturation at 95 °C for 2 min, followed by 40 cycles of denaturation at 95 °C for 15 s and annealing/elongation at 60 °C for 1 min. The melting curve was conducted at 95 °C for 20 min. The expression ratios of the genes of interest were analyzed by using the Lin-Reg PCR program (Heart Failure Research Center, Amsterdam, Netherlands).

### Biocompatibility test

Human urinary bladder T24 cells (ATCC^®^HTB-4^TM^) were used to test the biocompatibility of the active surface topographies. The glycerol stock of T24 cells was thawed in a water bath at 37 °C. Cells were quickly precipitated by centrifugation at 300 × *g* for 7 min and washed once with 2.1 mL prewarmed McCoy’s 5A medium supplemented with 10% Fetal Bovine Serum (FBS). The T24 cells were then transferred into 3 mL pre-warmed 10% FBS solution in a small petri-dish (with a diameter of 35 mm) and cultured at 37 °C supplemented with 5% CO_2_.

When the surface coverage on the bottom of the petri dish reached 80%, the spent FBS solution was replaced with fresh FBS solution. A sterile and insulated copper coil was placed directly onto the layer of T24 bladder cells and connected to a power source for generating a magnetic field at 1 or 5 mT. During actuation, the temperature change was measured using a temperature probe (Cole Parmer, Vernon Hills, IL, USA). After 3 h continuous actuation at 1 mT or 3 min on-demand actuation at 5 mT, cell viability was determined using LIVE/DEAD™ Viability/Cytotoxicity Kit for mammalian cells (ThermoFisher Scientific, Waltham, MA, USA) after a gentle wash with PBS.

### Nanoparticle release

To test if MNPs may leak from active PDMS pillars during actuation, Ferrozine assay^47^ was used to measure MNPs in the solution. First, a standard curve was established. To do this, dried MNPs were first suspended in PBS (pH 7.4) to obtain MNP suspension at concentrations of 0, 0.01, 0.1, 0.25, 0.5, or 1 mg mL^−1^. Then, MNPs solutions (50 µL) were dissolved with 1.4 M HCl (650 µL) at 65 °C for 2 h followed by overnight incubation in PBS at room temperature with shaking. The Fe^+^ released from the MNPs was diluted 40 times in solutions with 10 mM HCl and 50 mM NaOH before being transferred into a 96-well plate (180 µL in each well; *n* = 3 for each data point). With the addition of 20 µL of freshly made iron detection agent (containing 6.5 mM ferrozine, 6.5 mM neocuproine, 2.5 mM ammonium acetate, and 1 M ascorbic acid) and 30 min of shaking at room temperature, the standard curve was established based on the absorbance of each well measured at the wavelength of 562 nm using a plate reader (BioTek Epoch 2, Winooski, VT, USA; Supplementary Fig. 15). To understand the effects of pH on MNP release, the standard curves of MNPs at pH of 6, 7, and 7.4 were also established (Supplementary Fig. 15).

After the standard curves were established (Supplementary Fig. 15), PDMS pillars were continuously actuated in PBS with varying pH (6, 7, and 7.4) at 37 °C. Samples (50 µL) were taken at different time points during incubation, e.g., 0.5, 1, 1.5, 2, 3, 4, 6, 8, 12, 24, 48, and 96 h, and then processed as described above to determine the MNP release during incubation.

### Engineering a prototype catheter

PDMS surfaces with active topographies were prepared as described above and then fit into a 20 mm long rigid plastic tube as a mold with micron-sized pillars facing inside. The prototype was sealed with the same silicone elastomer (base:curing agent = 10:1). To evaluate the antifouling activities, the prototype catheters, static controls, and flat controls (no pillars) were connected into a flow cell system using luer locks. Biofilm formation was initiated by filling the prototype and control catheters with artificial urine medium^62^ inoculated with UPEC ATCC53505 to an OD_600_ of 0.05 and then letting it sit for 3 h. Then, artificial urine medium was flowed through the catheter continuously at 10 mL h^−1^.

To quantify the CFU of biofilms formed in the prototype catheters, static controls, and flat controls, the catheters were disconnected from the flow cell system. After gently washing the prototype catheters and controls once with sterile 0.85% NaCl solution, 0.1 g of 0.1 mm zirconia/silica beads were added and shaken for 1 min. The number of biofilm cells was quantified using the drop plate method, as described above.

### Statistics

One-way ANOVA and two-way ANOVA analyses followed by Tukey test were used for all statistical analyses using Windows version SAS 9.1.3 (SAS, Cary, NC, USA). The method based on one-way ANOVA is not mentioned repeatedly in the manuscript. The *p* values are shown in the Results section. Results with *p* < 0.05 were considered statistically significant.

### Reporting summary

Further information on research design is available in the Nature Research Reporting Summary linked to this article.

## Supplementary information

Supplementary Information

Description of Additional Supplementary Files

Supplementary Movie 1

Supplementary Movie 2

Reporting Summary

## Data Availability

In addition to Supplementary Information, data supporting the findings of this study are summarized in the Source Data file (10.6084/m9.figshare.12084171). Specifically, the source data underlying Figs. 1a, b, 2, 3c, 4a, b, 6b and Supplementary Figs. 2–6, 11, 12 are provided in the Source data file. Any other relevant data are available from the authors upon request.

## Source Data

Source Data

## Code availability

SAS codes for one-way and two-way ANOVA analyses and Tukey test are available from the authors upon request.
