# Supplementary material for: Magnetically driven active topography for long-term biofilm control
Source: Nat Commun. 2020 May 5;11:2211. doi: 10.1038/s41467-020-16055-5 (PMC7200660; doi:10.1038/s41467-020-16055-5)
Supplement: Supplementary file 1 — Supplementary Information [file 41467_2020_16055_MOESM1_ESM.pdf]

Supplementary Information

Magnetically driven active topography for long-term biofilm control

Gu, *et al*.

1. Supplementary Methods.

1.1 Predicting the deformation of active pillars during actuation. The deformation of pillars can be controlled by adjusting the strength of the electromagnetic field. Because the force was only applied at the tips of active pillars, we predicted their deformation (e_c) using the physical model that describes cantilever beam deflection[1] with one load applied at the end of a cylindrical stick [Supplementary Equation 1].

$$e_c = \mathrm{F} \cdot \frac{H_P^3}{3E_P(\pi\phi^2)} = m \cdot \mathbf{B} \cdot \frac{H_P^3}{3E_P(\pi\phi^2)} \qquad (1)$$

where F is the force generated by the movement of MNPs in response to external electromagnetic field; m is the magnetic moment of the particle; **B** is the applied electromagnetic field; H_P is the height; ϕ is the diameter of pillars; and E_P is the modulus of PDMS pillars (Fig. 1c). When the MNPs are secured in the tips of well-defined protruding features, both m and $\frac{H_P^3}{3E_P(\pi\phi^2)}$ are fixed (Supplementary Table 5), allowing us to program the deformation (e_c) by adjusting **B**.

1.2 Simulation of electric-current-induced heat generation. Actuation of the PDMS pillars in our design was powered by an electromagnetic field produced from solenoid coils. The temperature changes due to electric-current-induced heat were calculated using [Supplementary Equation 2]:

$$\mathrm{Q} \cdot \Delta t = (\mathrm{I}_i^2\mathrm{R}) \cdot \Delta t = \mathrm{C} \cdot \Delta\mathrm{T} \cdot \mathrm{m} \qquad (2)$$

where Q is the average power that is converted to heat (J s^{-1}); Δt is the length of actuation time (s); I_i is the electric current (A); R is the resistance of insulated copper coils (Ω); m is the mass of heat

conductive materials (g); C is the heat capacity of heat conductive materials (J g^{-1} °C^{-1}); and ΔT is the change in temperature (°C).

With a coil density (n) of 40 turns mm^{-1} and a current of 200 mA, the temperature change is estimated to be 8.8×10^{-4} °C per coil on the catheter surface (with a heat capacity around 0.17 J g^{-1} °C^{-1}) when the oscillation frequency is 10 Hz. If the catheter is inserted into a bladder of a human body with 80 kg and heat capacity of 3.475 J g^{-1} °C^{-1}, the temperature change in the surrounding tissue (~5 kg) would be around 7.9×10^{-9} °C. For a 3 or 6 h continuous actuation using a current of 40 mA, the temperature change in surrounding tissues is estimated to be 2.1×10^{-8} and 4.2×10^{-8} °C, respectively. Such effects on the viability of epithelial cells should be negligible.

1.3 Prediction of local magneto-thermal effects. We estimated the local heat generation from magneto-thermal effects using $E=m \cdot \mathbf{B}$,[2] in which E is the interaction energy magnitude of the iron-loaded ferritin configurations; m is the magnetic moment of the nanoparticles; and **B** is the strength of the magnetic field. Under our experimental condition (37 °C in a 5 mT), the maximum temperature change at the tip of active pillars due to magneto-thermal effect was estimated to be small as 6.1×10^{-5} °C per cycle of beating (even if there is absolutely no heat dissipation into the environment). Since the tips are in direct contact with liquid and moving with rapid heat dissipation, the level of heat generation is negligible.

2. Supplementary Tables

Supplementary Table 1. Biomass ($\mu m^3\ \mu m^{-2}$) of 48 h *P. aeruginosa* PAO1 biofilms on flat PDMS surfaces and PDMS surfaces with static or active surface topographies.

Surfaces \ Actuation mode	Continuous actuation for biofilm prevention (1 mT)	On-demand actuation for biofilm removal (5 mT)	Sequential combination of the two treatments
**Flat controls**	32.4 ±1.0		
**D = 2 µm**			
**Static controls**	20.8 ± 5.3		
**Active pillars**	0.06 ±0.007	1.9 ± 0.2	0.02 ± 0.07
***D = 5 µm**			
**Static controls**	21.9 ± 8.8		
**Active pillars**	0.01 ± 0.002	0.02 ± 0.002	0.01 ± 0.004
**D = 10 µm**			
**Static controls**	13.2 ± 2.2		
**Active pillars**	0.2 ± 0.1	0.1 ± 0.02	0.1 ± 0.02

*: the inter-pillar distance that demonstrated the optimal effects on the prevention and removal of *P. aeruginosa* PAO1 biofilms.

Supplementary Table 2. Biomass ($\mu m^3\ \mu m^{-2}$) of 48 h *S. aureus* ALC2085 biofilms on flat PDMS surfaces and PDMS surfaces with static or active surface topographies.

Surface \ Actuation mode	Continuous actuation for biofilm prevention (1 mT)	On-demand actuation for biofilm removal (5 mT)	Sequential combination of the two treatments
**Flat controls**	14.6 ± 1.5		
**D = 2 µm**			
**Static controls**	13.3 ± 2.3		
**Active pillars**	2.5 ± 0.6	0.7 ± 0.2	0.6 ± 0.1
***D = 5 µm**			
**Static controls**	15.2 ± 0.3		
**Active pillars**	0.3 ± 0.04	0.3 ± 0.02	0.1 ± 0.004
**D = 10 µm**			
**Static controls**	10.4 ± 2.5		
**Active pillars**	0.5 ±0.1	1.7 ± 1.1	0.3 ± 0.1

*: the inter-pillar distance that demonstrated the optimal effects on the prevention and removal of *S. aureus* ALC2085 biofilms.

Supplementary Table 3. Biomass (μm^3 μm^{-2}) of 48 h UPEC ATCC53505 biofilms on flat PDMS surfaces and PDMS surfaces with static or active surface topographies.

Surface \ Actuation mode	Continuous actuation for biofilm prevention (1 mT)	On-demand actuation for biofilm removal (5 mT)	Sequential combination of the two treatments
**Flat controls**	9.2 ± 3.0		
**D = 2 µm**			
**Static controls**	7.5 ± 0.3		
**Active pillars**	0.02 ± 0.01	0.01 ± 0.006	0.008 ± 0.01
***D = 5 µm**			
**Static controls**	10.1 ± 4.4		
**Active pillars**	0.002 ± 0.0003	0.002 ± 0.002	0.0004 ± 0.0003
**D = 10 µm**			
**Static controls**	2.6 ± 0.7		
**Active pillars**	0.002 ± 0.0005	0.0003 ± 0.0001	0.0002 ± 0.0001

*: the inter-pillar distance that demonstrated the optimal effects on the prevention and removal of UPEC ALC53505 biofilms.

Supplementary Table 4. Primers for qPCR.

Genes	Primer sequence (5’-3’)
*rrs*A [housekeeping (HK) gene][3]	Forward primer: CTCTTGCCATCGGATGTGCCCA
	Reverse primer: CAGTGTGGCTGGTCATCCTCTCA
*rrs*B (16s rRNA)	Forward primer: GCGGACGGGTGAGTAATGTC
	Reverse primer: GTCCCCCTCTTTGGTCTTGC
*rrl*B (23s rRNA)	Forward primer: ACGGAGAAGGCTATGTTGGC
	Reverse primer: GTGTCGGTTTGGGGTACGAT
*rrn*B P1 (spacer-1 region)	Forward primer: CGGTTCACCGTTGCTCGAT
	Reverse primer: GGCGTCCCAACTTTGGCTA

Supplementary Table 5. Parameters used for simulation.

Parameters	Values	Source
MNP size	12.5 ± 4.7 nm	This study
MNP magnetic properties (m)	76 emu/g (measured under 18 kOe at room temperature)	Salaza, *et al.* [4]
Pillar height (H_P)	10 μm	This study
Pillar diameter (ϕ)	2 μm	This study
Young's modulus of pillars (E_P; base: cure agent = 10: 1)	2.1 ± 0.1 MPa	Previous study of our group[5]

3. Supplementary Figures

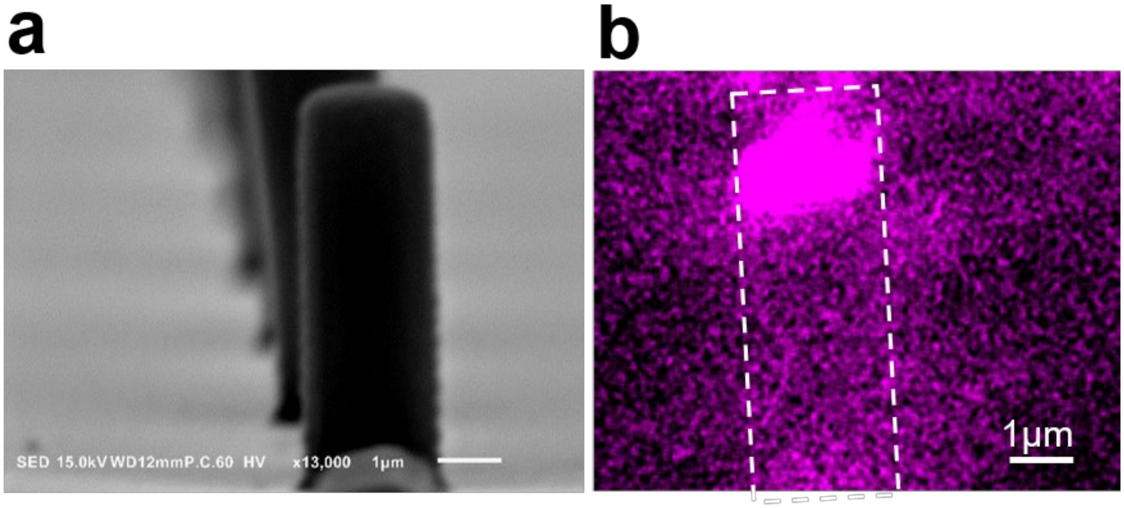

Supplementary Figure 1. Mapping the distribution of MNPs in PDMS pillars using SEM-EDS. Detection of Fe (atom) in the pillars using SEM-EDS. (a) SEM images of PDMS pillars. (b) EDS result showing the distribution of Fe signals (in purple). The profile of a pillar is highlighted with white dotted lines. Five images were randomly taken from each sample and two biological replicates were analyzed (n=2).

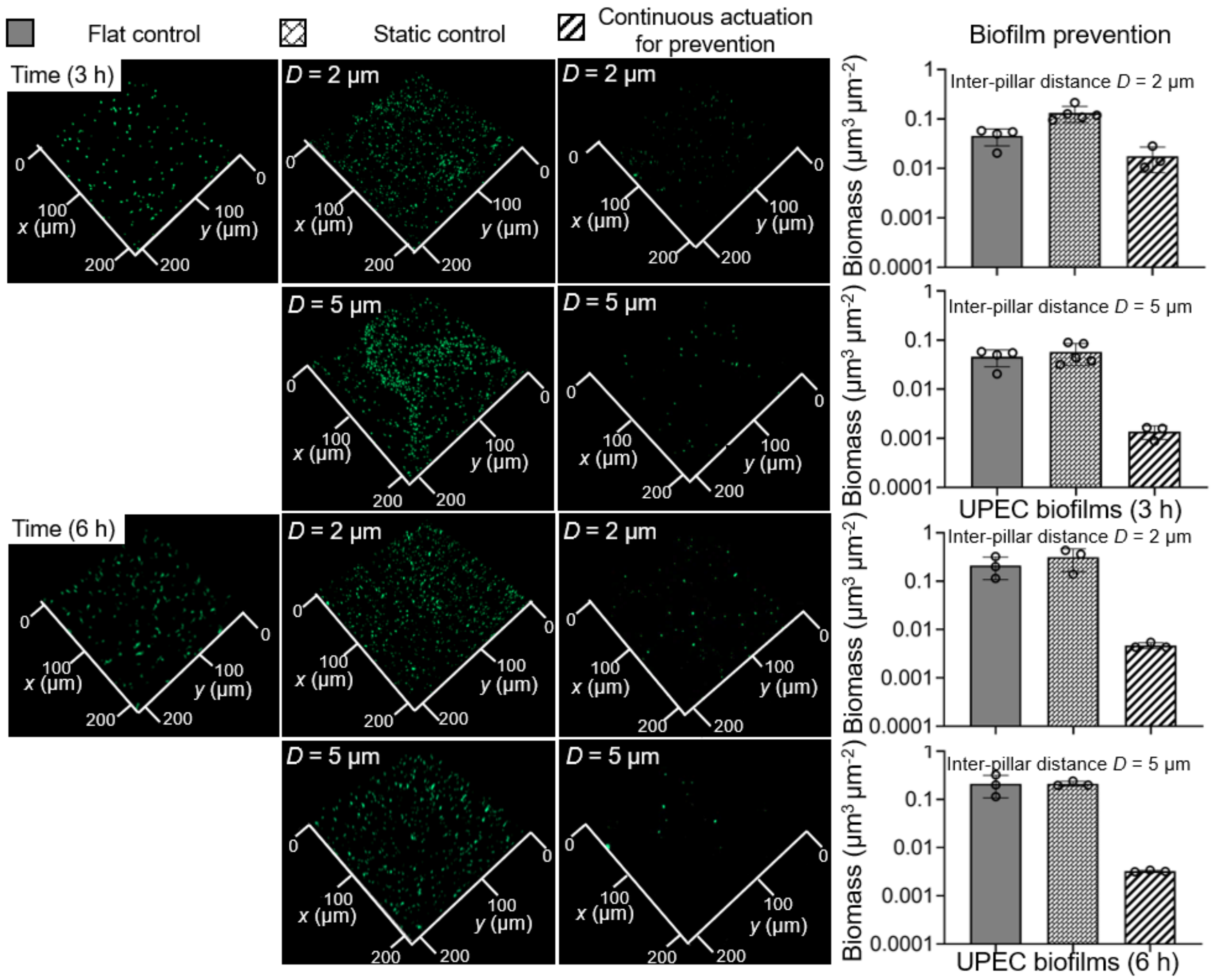

Supplementary Figure 2. Active pillars inhibited the early-stage biofilm formation of UPEC ATCC53505. Representative fluorescence images of biofilms on flat controls, static controls, and PDMS surfaces with active surface topographies are shown. The pillars were 10 µm tall with a diameter of 2 µm and inter-pillar distance of 2 or 5 µm. Active surface topographies were operated with a continuous beating for biofilm prevention. Biofilms were formed in static LB medium for 3 and 6 h and gently washed before being labeled with STYO®9 (green fluorescence). The biomass of UPEC ATCC53505 cells was quantified using COMSTAT[6]. The samples in the bar graph are indicated with the patterned labels above the corresponding fluorescence images. Each condition was tested with at least three biological replicates (error bar = standard deviation; n = 3-5), and

five random images were taken from each sample. Source data of Supplementary Fig. 2 are provided in the Source Data file.

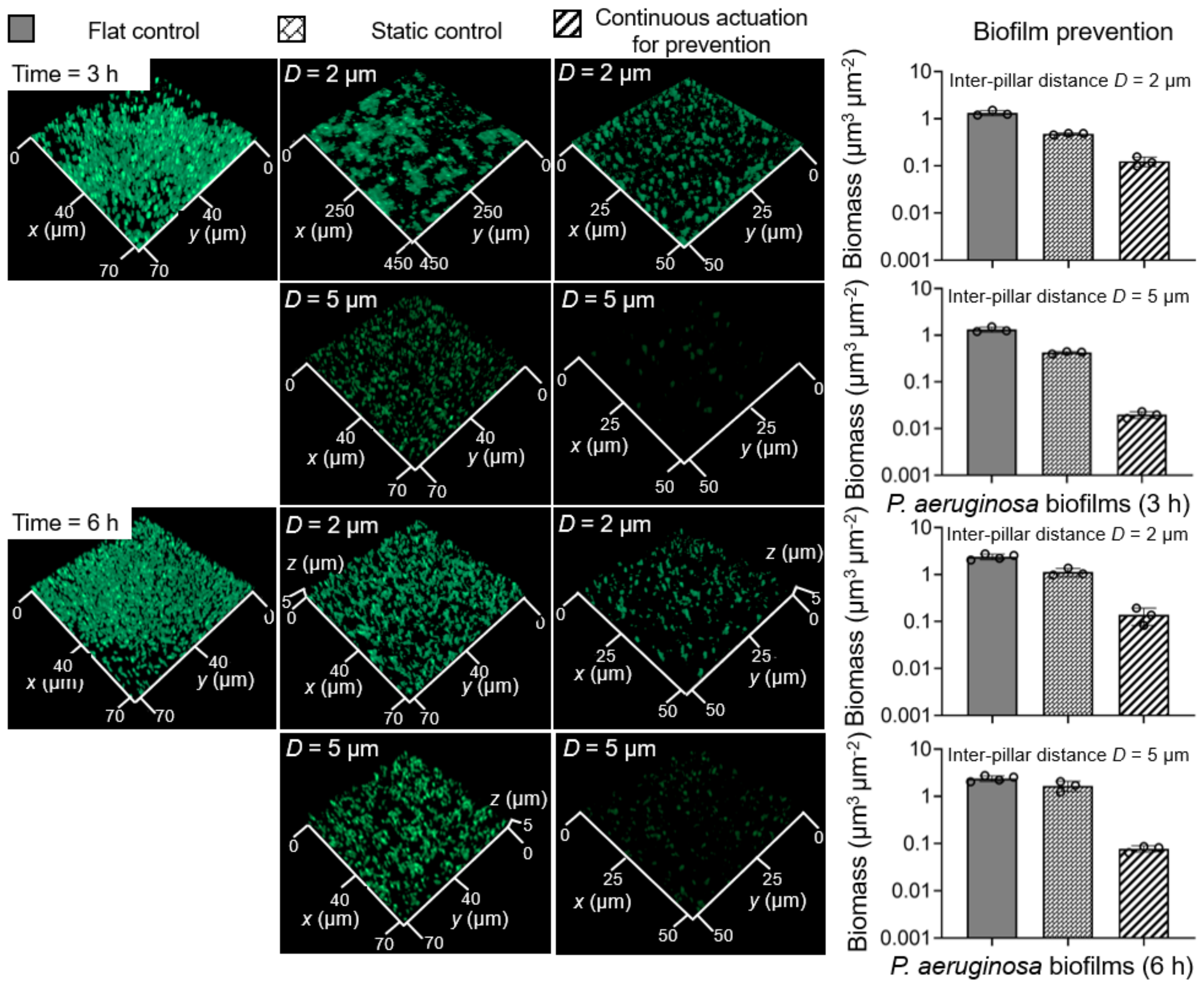

Supplementary Figure 3. Active pillars inhibited the early-stage biofilm formation of *P. aeruginosa* PAO1. Representative fluorescence images of biofilms on flat controls, static controls, and PDMS surfaces with active surface topographies are shown. The pillars were 10 µm tall with a diameter of 2 µm and inter-pillar distance of 2 or 5 µm. Active surface topographies were operated with a continuous beating for biofilm prevention. Biofilms were formed in static LB medium for 3 and 6 h and gently washed before being labeled with STYO®9 (green fluorescence). The biomass of *P. aeruginosa* PAO1 cells was quantified using COMSTAT[6]. The samples in the bar graph are indicated with the patterned labels above the corresponding fluorescence images. Each condition was tested with at least three biological replicates (error bar = standard deviation;

n = 3-4), and five random images were taken from each sample. Source data of Supplementary Fig. 3 are provided in the Source Data file.

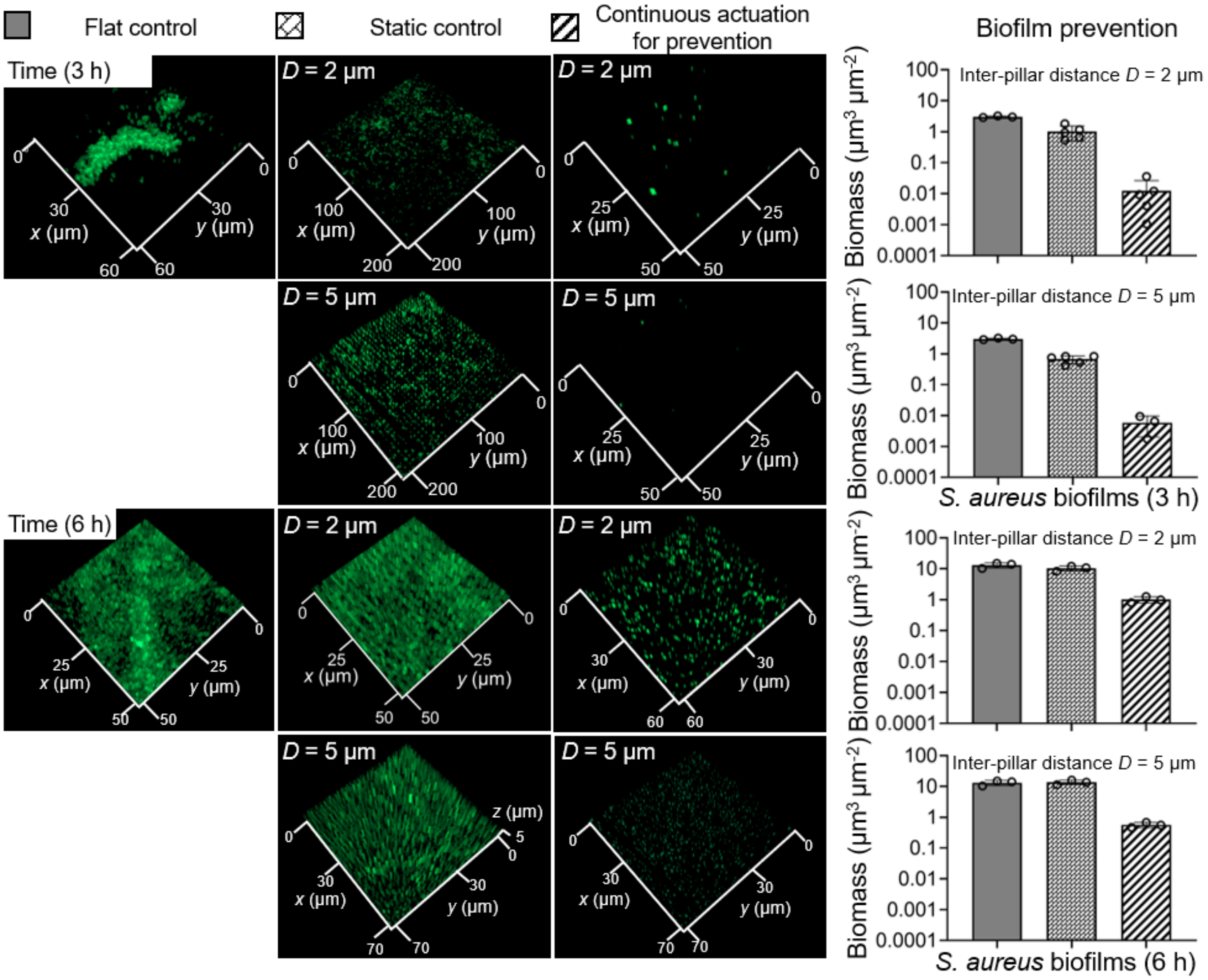

Supplementary Figure 4. Active pillars inhibited the early-stage biofilm formation of *S. aureus* ALC2085. Representative fluorescence images of biofilms on flat controls, static controls, and PDMS surfaces with active surface topographies are shown. The pillars were 10 μm tall with a diameter of 2 μm and inter-pillar distance of 2 or 5 μm. Active surface topographies were operated with a continuous beating for biofilm prevention. Biofilms were formed in static LB medium for 3 and 6 h and gently washed before being labeled with STYO®9 (green fluorescence). The biomass of *S. aureus* ALC2085 cells was quantified using COMSTAT[6]. The samples in the bar graph are indicated with the patterned labels above the corresponding fluorescence images. Each condition was tested with at least three biological replicates (error bar = standard deviation;

n = 3-5), and five random images were taken from each sample. Source data of Supplementary Fig. 4 are provided in the Source Data file.

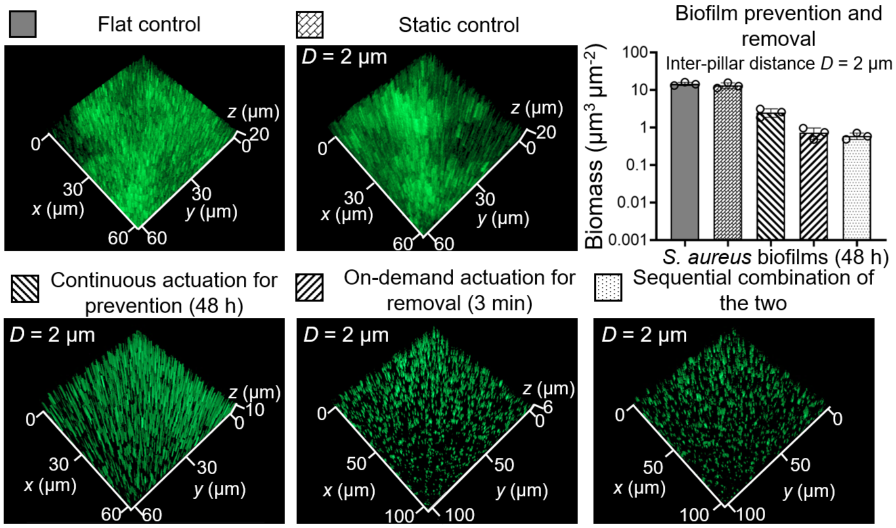

Supplementary Figure 5. Antifouling effects of active pillars against *S. aureus* ALC2085 biofilms. Representative fluorescence images of biofilms on flat controls, static controls, and PDMS surfaces with active surface topographies are shown. The pillars were 10 µm tall with a diameter of 2 µm and inter-pillar distance of 2 µm. Active surface topographies were operated in three modes for biofilm prevention and removal, including continuous beating for biofilm prevention, on-demand removal of mature biofilms (only actuated for 3 min after 48 h biofilm growth), and a sequential combination of these two treatments. Biofilms were formed in static LB medium for 48 h and gently washed before being labeled with STYO®9 (green fluorescence). The biomass of *S. aureus* ALC2085 cells was quantified using COMSTAT[6]. The samples in the bar graph are indicated with the pattern labels above the corresponding fluorescence images. Each condition was tested with three biological replicates (error bar = standard deviation; n = 3), and five random images were taken from each sample. Source data of Supplementary Fig. 5 are provided in the Source Data file.

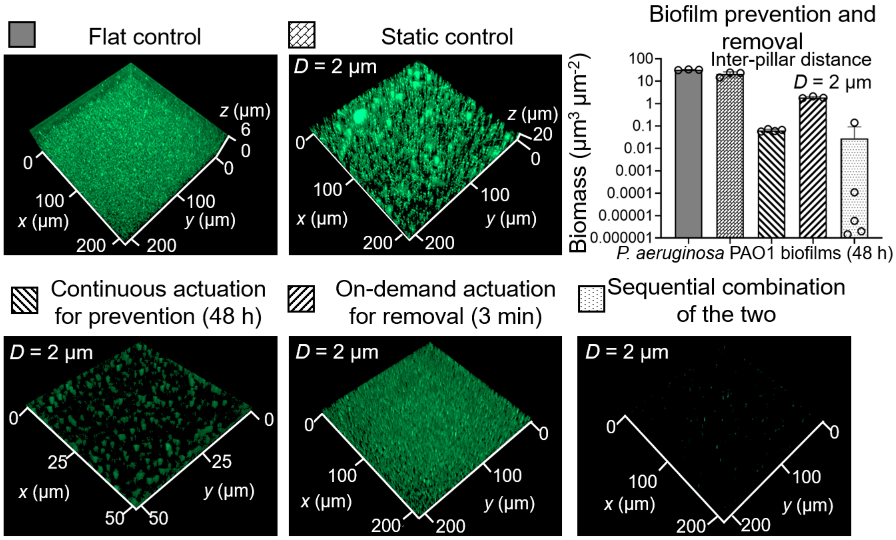

Supplementary Figure 6. Antifouling effects of active pillars against *P. aeruginosa* PAO1 biofilms. Representative fluorescence images of biofilms on flat controls, static controls, and PDMS surfaces with active surface topographies are shown. The pillars were 10 µm tall with a diameter of 2 µm and inter-pillar distance of 2 µm. Active surface topographies were operated in three modes for biofilm prevention and removal, including continuous beating for biofilm prevention, on-demand removal of mature biofilms (only actuated for 3 min after 48 h of biofilm growth), and a sequential combination of these two treatments. Biofilms were formed in static LB medium for 48 h and gently washed before being labeled with STYO®9 (green fluorescence). The biomass of *P. aeruginosa* PAO1 cells was quantified using COMSTAT[6]. The samples in the bar graph are indicated with the pattern labels above the corresponding fluorescence images. Each condition was tested with at least three biological replicates (error bar = standard deviation; n = 3-5), and five random images were taken from each sample. Source data of Supplementary Fig. 6 are provided in the Source Data file.

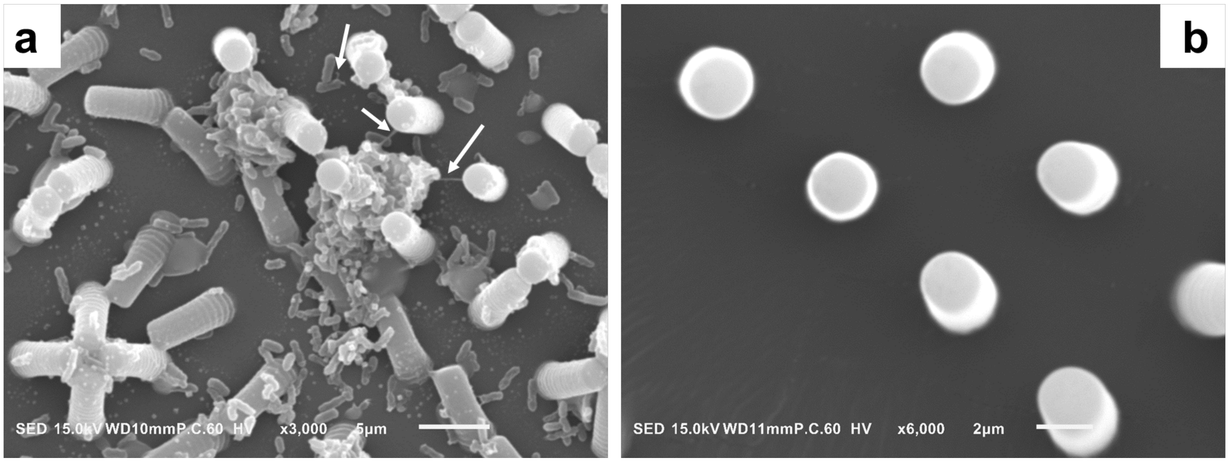

Supplementary Figure 7. Effects of on-demand actuation on mature UPEC biofilms. Representative SEM images of 48 h UPEC biofilms on PDMS surfaces before (a) and after (b) 3-min on-demand actuation. The pillars were 10 µm tall with a diameter of 2 µm and inter-pillar distance of 5 µm. Some extracellular structures in the SEM images before actuation are highlighted with white arrows. Five images were randomly taken from each sample and two biological replicates were analyzed (n=2).

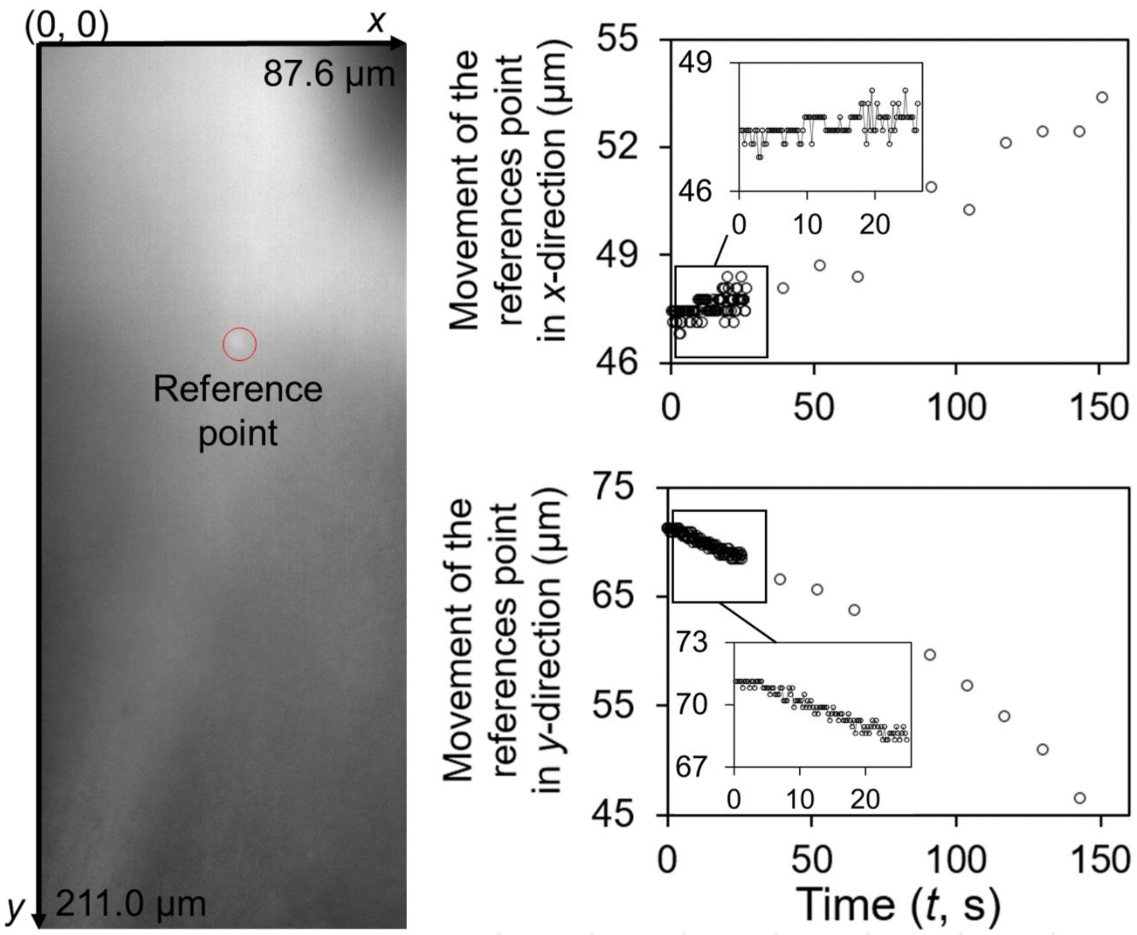

Supplementary Figure 8. On-demand actuation induced structural changes in *P. aeruginosa* PAO1 biofilms. The pillars were 10 μm tall with a diameter of 2 μm and inter-pillar distance of 2 μm. The 48 h *P. aeruginosa* PAO1 biofilm was labeled with SYTO® 9. A time-lapse movie was taken with a 0.26 s time interval. We tracked the movement of the reference point in both *x* and *y*-directions every 0.26 s during the first 26 s and every 13 s for the rest of the 3-min on-demand actuation. Active pillars beat along the *x*-axis. The tracking showed that the reference point had propagation in both *x* and *y*-directions. After the 3-min propagation, the reference point exhibited more movement in the *y*-direction (26.8 μm) compared to the *x*-direction (5.9 μm).

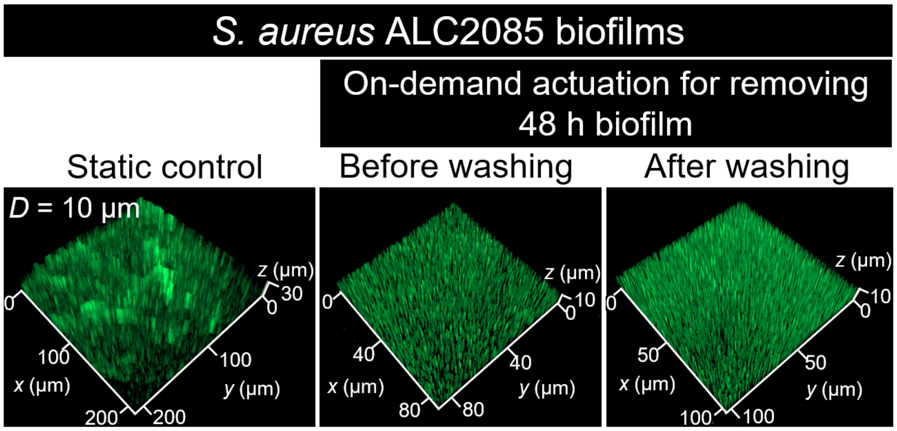

Supplementary Figure 9. Effects of on-demand actuation on mature *S. aureus* biofilms. Representative fluorescence images of 48 h *S. aureus* ALC2085 biofilms on PDMS surfaces with active pillars (D = 10 μm) before and after 3 min on-demand actuation (5 mT; with or without wash). Each condition was tested with three biological replicates (n = 3), and five random images were taken from each sample.

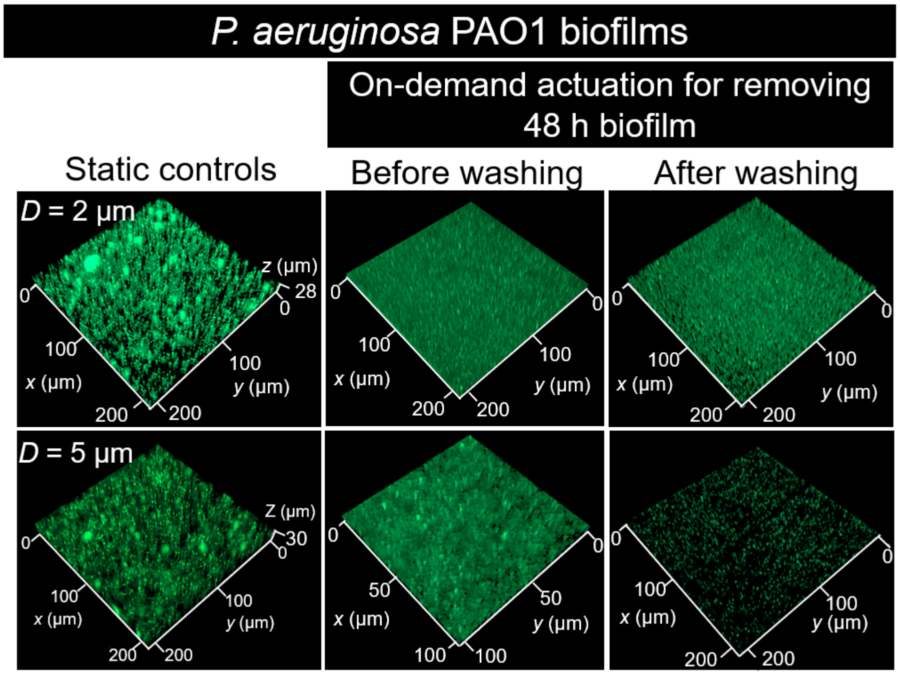

Supplementary Figure 10. Effects of on-demand actuation on mature *P. aeruginosa* PAO1 biofilms. Representative fluorescence images of 48 h *P. aeruginosa* PAO1 biofilms on PDMS surfaces with active pillars (*D* = 2 and 5 µm) before and after 3 min on-demand actuation (5 mT; with or without washing). The static controls and samples after washing with *D* = 2 µm have been shown in Supplementary Fig. 6. They are repeated here for comparison to show the effects of pillar spacing and wash. Each condition was tested with three biological replicates (n = 3), and five random images were taken from each sample.

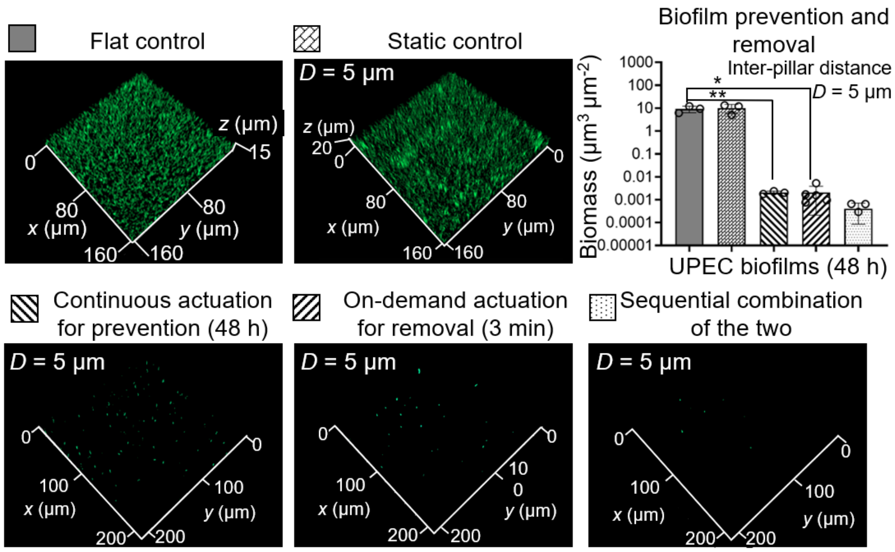

Supplementary Figure 11. Active pillars exhibited profound antifouling effects against UPEC biofilms. Representative fluorescence images of biofilms on flat controls, static controls, and PDMS surfaces with active surface topographies are shown. The pillars were 10 μm tall with a diameter of 2 μm and inter-pillar distance of 5 μm. Active surface topographies were operated in three modes for biofilm prevention and removal, including continuous beating for biofilm prevention, on-demand removal of mature biofilms (only actuated for 3 min after 48 h biofilm growth), and a sequential combination of these two treatments. Biofilms were formed in static LB medium for 48 h and gently washed before being labeled with STYO®9 (green fluorescence). The biomass of UPEC ATCC53505 cells was quantified using COMSTAT[6]. The samples in the bar graph are indicated with the pattern labels above the corresponding fluorescence images. Each condition was tested with at least three biological replicates (error bar = standard deviation; n = 3-5; * $p = 0.0057$ and ** $p = 0.0003$, respectively, one-way ANOVA adjusted by Tukey test), and five random images were taken from each sample. Source data of Supplementary Fig. 11 are provided in the Source Data file.

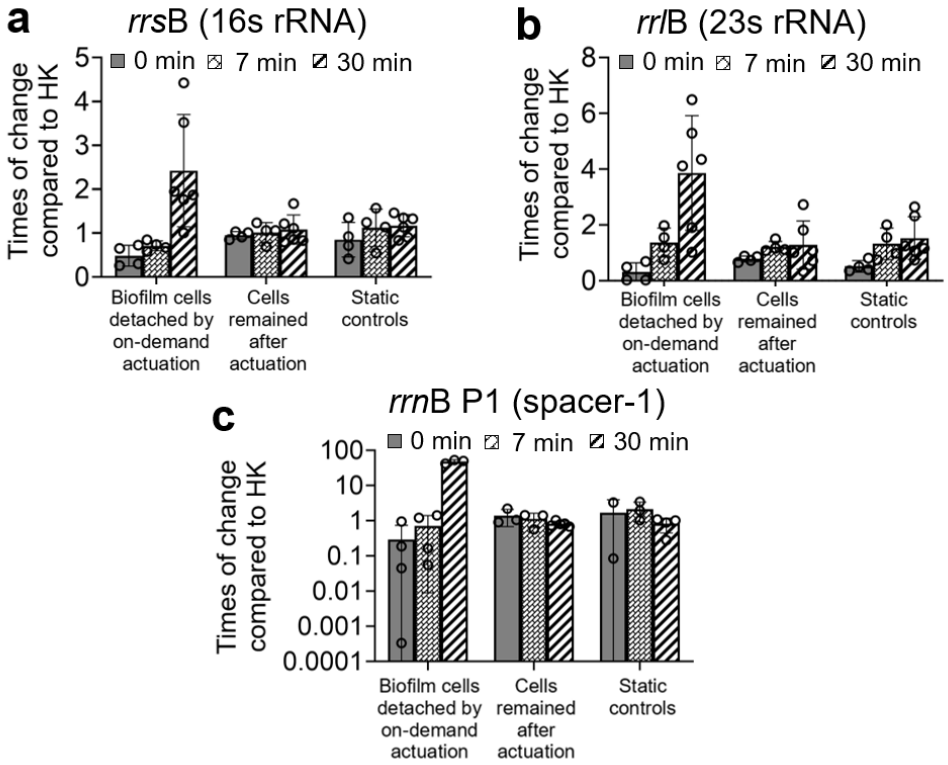

Supplementary Figure 12. On-demand actuation triggered changes in mature UPEC biofilm cells. Expression levels of three genes, including *rrs*B (a), *rrl*B (b), and *rrn*B P1 (c), were compared. The housekeeping gene is *rrs*A[3]. Biofilm cells were harvested via two different methods, including 3-min on-demand actuation and bead beating (for remaining biofilm cells after actuation and biofilms formed on static controls without actuation). The biofilm cells dispersed by beat beating and then treated with an antibiotic are referred to as Static controls. To avoid the confounding effects of bead beating, cells dispersed by actuation were also subjected to bead beating. Each condition was tested with at least three biological replicates (error bar = standard deviation; n = 3-6). Source data of Supplementary Fig. 12 are provided in the Source Data file.

a

b

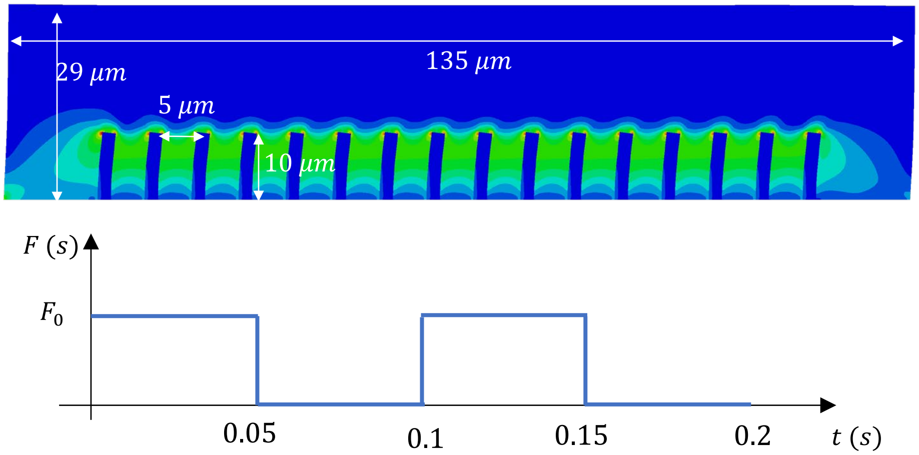

Supplementary Figure 13. Distribution of principal strain in mature biofilms during on-demand actuation. The inter-pillar distance was 5 μm, and biofilm thickness was 29 μm. (a) A simulation snapshot shows the pillar beating in a biofilm matrix. The color represents the maximum principal strain at time = 0.05 s. (b) The amplitude of force applied to the pillars.

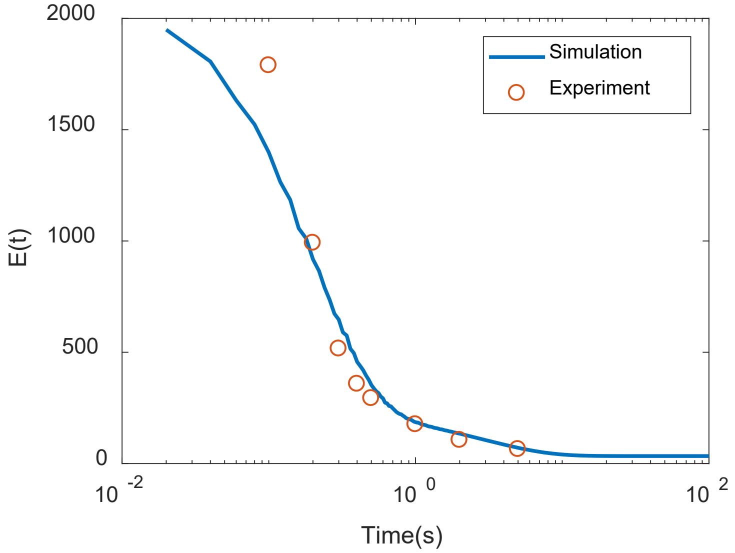

Supplementary Figure 14. FEM simulations to predict the viscoelasticity of *P. aeruginosa* biofilms. The graphs show the normalized stress in compressed biofilms (a strain of 0.1) as a function of relaxation time from FEM simulation and experiments[7].

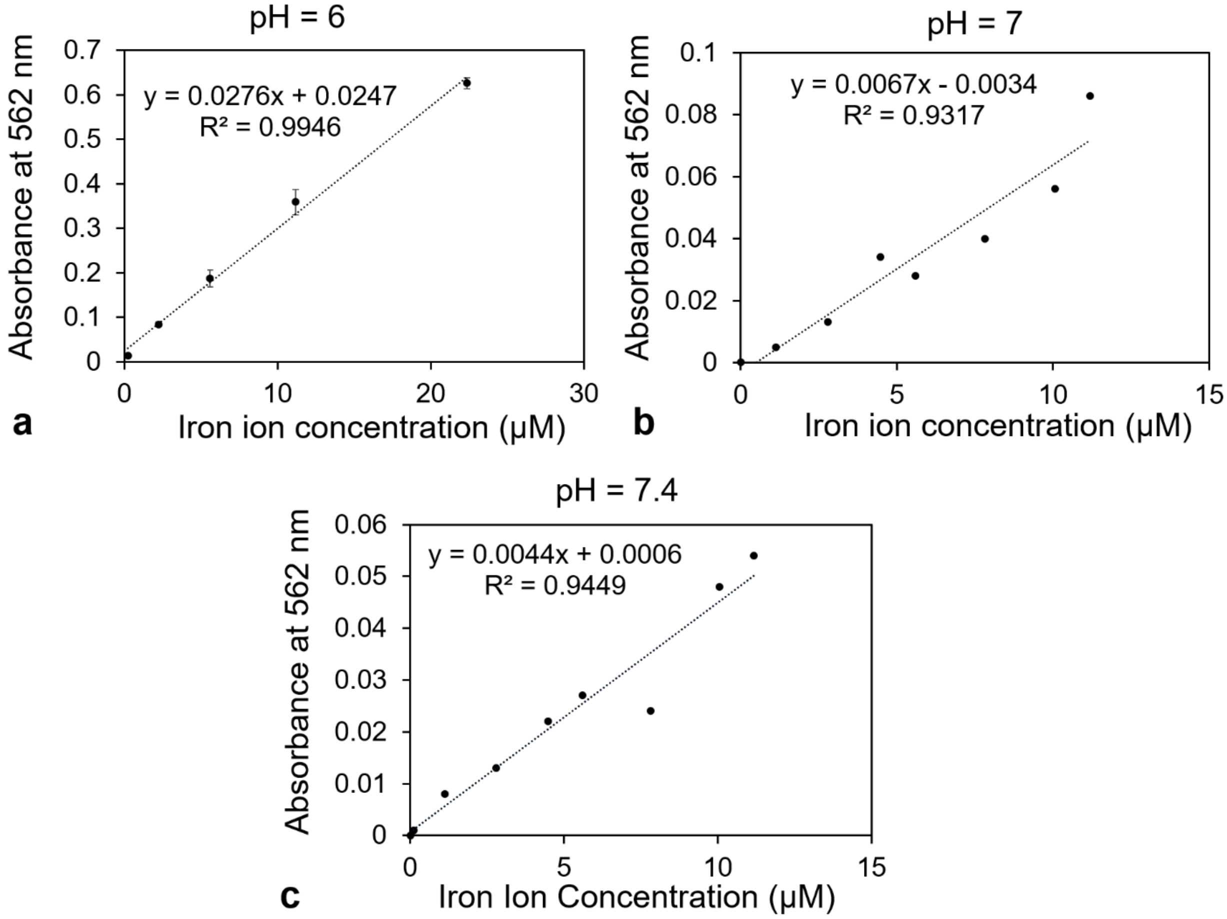

Supplementary Figure 15. Standard curves of the Ferrozine assay. (a-c) Standard curves of the Fe^{3+} in PBS with different pH [6 (a), 7 (b), or 7.4 (c)] at 37 °C.

4. Supplementary References

1 Belendez, T., Neipp, C. & Belendez, A. Large and small deflections of a cantilever beam. *Eur. J. Phys.* **23**, 371-379, doi: 10.1088/0143-0807/23/3/317 (2002).

2 Barbic, M. Possible magneto-mechanical and magneto-thermal mechanisms of ion channel activation in magnetogenetics. *Elife* **8**, doi:10.7554/eLife.45807 (2019).

3 Zhou, K., Zhou, L., Lim, Q'., Zou, R., Stephanopoulos, G., Too, H.P.. Novel reference genes for quantifying transcriptional responses of *Escherichia coli* to protein overexpression by quantitative PCR. *BMC Mol. Biol.* **12**, 18, doi:10.1186/1471-2199-12-18 (2011).

4 Salazar, J. S., Perez, L., de Abril, O., Phuoc, L. T., Ihiawakrim, D., Vazquez, M., Greneche, J. M., Begin-Colin, S., and Pourroy, G.. Magnetic iron oxide nanoparticles in 10−40 nm range: Composition in terms of magnetite/maghemite ratio and effect on the magnetic properties. *Chem. Mater.* **23**, 1379-1386, doi: 10.1021/cm103188a (2011).

5 Song, F. and Ren, D. Stiffness of cross-linked poly(dimethylsiloxane) affects bacterial adhesion and antibiotic susceptibility of attached cells. *Langmuir* **30**, 10354-10362, doi:10.1021/la502029f (2014).

6 Heydorn, A., Nielsen, A.T., Hentzer, M., Sternberg, C., Givskov, M., Ersbøll, B.K., and Molin, S. Quantification of biofilm structures by the novel computer program COMSTAT. *Microbiology* **146 (Pt 10)**, 2395-2407, doi: 10.1099/00221287-146-10-2395 (2000).

7 Rozenbaum, R.T., van der Mei, H.C., Woudstra, W., de Jong, E.D., Busscher, H.J., and Sharma, P.K. Role of viscoelasticity in bacterial killing by antimicrobials in differently grown. *Antimicrob. Agents Chemother.* **63**, e01972-18, doi:10.1128/AAC.01972-18 (2019).
