# Supplementary material for: Magnetically driven active topography for long-term biofilm control
Source: Nat Commun. 2020 May 5;11:2211. doi: 10.1038/s41467-020-16055-5 (PMC7200660; doi:10.1038/s41467-020-16055-5)
Supplement: Supplementary file 2 — Description of Additional Supplementary Files [file 41467_2020_16055_MOESM2_ESM.docx]

Description of Additional Supplementary Files

**Supplementary Movie 1**. Time-lapse movie of a 48 h UPEC ATCC53505 biofilm during ondemand actuation. The movie was recorded with a 4.28 s time interval between frames. Biofilms were formed in static LB medium for 48 h and gently washed before being labeled with STYO®9 (green fluorescence). Active pillars beat left to right. The pillars were 10 µm tall with a diameter of 2 µm and inter-pillar distance of 5 µm. Bar = 20 µm. (DOI: 10.6084/m9.figshare.12049194)

**Supplementary Movie 2.** Time-lapse movie of a 48 h P. aeruginosa PAO1 biofilm during ondemand actuation. The movie was recorded with a 0.26 s time interval between frames, and edited to show images with a 13 s time interval (between every 50 frames). In this movie, the reference point is highlighted with a red circle. Active pillars beat left to right. The pillars were 10 µm tall with a diameter of 2 µm and inter-pillar distance of 2 µm. The tracking of the reference point showed propagation in both the x and y-directions. After the 3-min propagation, the reference point exhibited a bigger movement in the y-direction (26.8 µm) compared to the x-direction (5.9 µm). Bar = 20 µm. (DOI: 10.6084/m9.figshare.12049200)
