# Supplementary material for: Magnetically driven active topography for long-term biofilm control
Source: Nat Commun. 2020 May 5;11:2211. doi: 10.1038/s41467-020-16055-5 (PMC7200660; doi:10.1038/s41467-020-16055-5)
Supplement: Supplementary file 5 — Reporting Summary [file 41467_2020_16055_MOESM5_ESM.pdf]

nature research

Corresponding author(s): Dacheng Ren

Last updated by author(s): Feb 20, 2020

Reporting Summary

Nature Research wishes to improve the reproducibility of the work that we publish. This form provides structure for consistency and transparency in reporting. For further information on Nature Research policies, see Authors & Referees and the Editorial Policy Checklist .

Statistics

For all statistical analyses, confirm that the following items are present in the figure legend, table legend, main text, or Methods section.

n/a	Confirmed	
☐	☒	The exact sample size (*n*) for each experimental group/condition, given as a discrete number and unit of measurement
☐	☒	A statement on whether measurements were taken from distinct samples or whether the same sample was measured repeatedly
☐	☒	The statistical test(s) used AND whether they are one- or two-sided<br>*Only common tests should be described solely by name; describe more complex techniques in the Methods section.*
☐	☒	A description of all covariates tested
☐	☒	A description of any assumptions or corrections, such as tests of normality and adjustment for multiple comparisons
☐	☒	A full description of the statistical parameters including central tendency (e.g. means) or other basic estimates (e.g. regression coefficient) AND variation (e.g. standard deviation) or associated estimates of uncertainty (e.g. confidence intervals)
☐	☒	For null hypothesis testing, the test statistic (e.g. *F*, *t*, *r*) with confidence intervals, effect sizes, degrees of freedom and *P* value noted<br>*Give P values as exact values whenever suitable.*
☒	☐	For Bayesian analysis, information on the choice of priors and Markov chain Monte Carlo settings
☒	☐	For hierarchical and complex designs, identification of the appropriate level for tests and full reporting of outcomes
☒	☐	Estimates of effect sizes (e.g. Cohen's *d*, Pearson's *r*), indicating how they were calculated

Our web collection on statistics for biologists contains articles on many of the points above.

Software and code

Policy information about availability of computer code

Data collection: In this study, we used Matlab 2015 based COMSTAT to quantify biofilm biomass based on the fluorescent signals from 3D biofilm images. COMSTAT is an open-source code kindly provided by Dr. Arne Heydorn at the Technical University of Denmark and also available at the website http://www.comstat.dk/.

Data analysis: In this study, we have used a commercially available software ABAQUS/Standard to simulate the stress generated by the beating of active pillars in viscoelastic materials. For statistics, we used another standard software, SAS, to conduct the one-way and two way ANOVA and Tukey test.

For manuscripts utilizing custom algorithms or software that are central to the research but not yet described in published literature, software must be made available to editors/reviewers. We strongly encourage code deposition in a community repository (e.g. GitHub). See the Nature Research guidelines for submitting code & software for further information.

Data

Policy information about availability of data

All manuscripts must include a data availability statement. This statement should provide the following information, where applicable:

- Accession codes, unique identifiers, or web links for publicly available datasets
- A list of figures that have associated raw data
- A description of any restrictions on data availability

Data Availability. In addition to Supplementary Information, data supporting the findings of this study are summarized in the Source Data file (DOI: 10.6084/m9.figshare.12084171). Specifically, the source data underlying Figs. 1a&b, 2, 3c, 4a&b, 6b and Supplementary Figs 2-6, 11, 12 are provided in the Source Data file. Any other relevant data are available from the authors upon request.

Field-specific reporting

Please select the one below that is the best fit for your research. If you are not sure, read the appropriate sections before making your selection.

- [x] Life sciences
- [ ] Behavioural & social sciences
- [ ] Ecological, evolutionary & environmental sciences

For a reference copy of the document with all sections, see nature.com/documents/nr-reporting-summary-flat.pdf

Life sciences study design

All studies must disclose on these points even when the disclosure is negative.

Sample size	This is an in vitro study. Each condition was tested with at least three biological replicates, which is standard in this field [e.g., Levering and Lopez, et al. Urinary catheter capable of repeated on-demand removal of infectious biofilms via active deformation. Biomaterials 7, 77-86, (2016)]. We have indicated the number of repeats in the related figures and discussion.
Data exclusions	All raw data are included in the analysis. No data were excluded.
Replication	As described above, each condition was repeated with at least three biological replicates. Some experiments failed because of contaminations or accidental sample damage. We have included the data of all qualified experimental results to avoid bias. The number of repeats (N) has been indicated in related figures and text.
Randomization	The samples were from independent biological repeats. The order of sample analysis was randomized.
Blinding	This is not a clinical trial. However, the samples were mixed and tested in a randomized order to avoid bias. We also conducted blinded experiments as much as possible (such as imaging, qPCR and cell viability test), in which the samples were prepared and labeled by one person based on sample type and analyzed by different researchers in a blinded manner.

Reporting for specific materials, systems and methods

We require information from authors about some types of materials, experimental systems and methods used in many studies. Here, indicate whether each material, system or method listed is relevant to your study. If you are not sure if a list item applies to your research, read the appropriate section before selecting a response.

Materials & experimental systems

n/a	Involved in the study	
x		Antibodies
	x	Eukaryotic cell lines
x		Palaeontology
x		Animals and other organisms
x		Human research participants
x		Clinical data

Methods

n/a	Involved in the study	
x		ChIP-seq
x		Flow cytometry
x		MRI-based neuroimaging

Eukaryotic cell lines

Policy information about cell lines

Cell line source(s)	Human urinary bladder T24 (ATCC® HTB-4)
Authentication	This cell line was not authenticated. However, we monitored the cells under the microscope Zeiss observer Z1 to check the morphology of the cells. They look uniform and free of contamination or microbes.
Mycoplasma contamination	Since this is a standard cell line from ATCC, and we did not observe any difference in growth and activities among cells (signature characters of mycoplasma contamination), we did not conduct any further specific tests.
Commonly misidentified lines (See ICLAC register)	No commonly misidentified cell lines were used in the study.
